# Supplementary material for: Excess weight is associated with neurological and neuropsychiatric symptoms in post-COVID-19 condition: A systematic review and meta-analysis
Source: PLoS One. 2025 May 7;20(5):e0314892. doi: 10.1371/journal.pone.0314892 (PMC12057935; doi:10.1371/journal.pone.0314892)
Supplement: S1 Table — (DOC) [file pone.0314892.s001.doc]

**Supporting Information**

**S1 Table. Database search strategy.**

| **Database** | **Search** (July 3^rd^ , 2023) |
| --- | --- |
| PUBMED | **(((((((((((((((((((((((((((((((((((((((((((((((((((((((((((((((((((((((((((((((((((((((((((Post acute covid 19 syndrome[MeSH Terms]) OR (COVID-19[MeSH Terms])) OR (SARS-CoV-2[MeSH Terms])) OR (Long COVID-19[Title/Abstract])) OR (Post acute covid 19 syndrome[Title/Abstract])) OR (COVID-19[Title/Abstract])) OR (SARS-CoV-2[Title/Abstract])) OR (2019 nCoV Disease[Title/Abstract])) OR (2019 nCoV Infection[Title/Abstract])) OR (2019 Novel Coronavirus Disease[Title/Abstract])) OR (2019 Novel Coronavirus Infection[Title/Abstract])) OR (2019 Novel Coronavirus*[Title/Abstract])) OR (2019-nCoV[Title/Abstract])) OR (2019-nCoV Disease*[Title/Abstract])) OR (2019-nCoV Infection*[Title/Abstract])) OR (Coronavirus 2, SARS[Title/Abstract])) OR (Coronavirus Disease 19[Title/Abstract])) OR (Coronavirus Disease 2019[Title/Abstract])) OR (Coronavirus Disease 2019 Virus[Title/Abstract])) OR (Coronavirus Disease-19[Title/Abstract])) OR (Coronavirus, 2019 Novel[Title/Abstract])) OR (Coronavirus, Wuhan[Title/Abstract])) OR (COVID 19[Title/Abstract])) OR (COVID 19 Virus[Title/Abstract])) OR (COVID 19 Pandemic[Title/Abstract])) OR (COVID 19 Virus Disease[Title/Abstract])) OR (COVID 19 Virus Infection[Title/Abstract])) OR (COVID, Long-Haul[Title/Abstract])) OR (COVID19[Title/Abstract])) OR (COVID-19 Syndrome, Post-Acute[Title/Abstract])) OR (COVID19 Virus*[Title/Abstract])) OR (COVID-19 Pandemic*[Title/Abstract])) OR (COVID-19 Virus Disease*[Title/Abstract])) OR (COVID-19 Virus Infection*[Title/Abstract])) OR (COVID-19, Long Haul[Title/Abstract])) OR (Disease 2019, Coronavirus[Title/Abstract])) OR (Disease, 2019-nCoV[Title/Abstract])) OR (Disease, COVID-19 Virus[Title/Abstract])) OR (Infection, 2019-nCoV[Title/Abstract])) OR (Infection, COVID-19 Virus[Title/Abstract])) OR (Infection, SARS-CoV-2[Title/Abstract])) OR (Long COVID[Title/Abstract])) OR (Long Haul COVID 19*[Title/Abstract])) OR (Long Haul COVID*[Title/Abstract])) OR (Long Haul COVID-19[Title/Abstract])) OR (Long-Haul COVID[Title/Abstract])) OR (Novel Coronavirus, 2019[Title/Abstract])) OR (Pandemic, COVID-19[Title/Abstract])) OR (Post Acute COVID 19 Syndrome[Title/Abstract])) OR (Post Acute COVID-19 Syndrome*[Title/Abstract])) OR (Post Acute Sequelae of SARS CoV 2 Infection[Title/Abstract])) OR (Post COVID Conditions[Title/Abstract])) OR (Post-Acute Sequelae of SARS-CoV-2 Infection[Title/Abstract])) OR (Post-COVID Condition*[Title/Abstract])) OR (SARS Coronavirus 2[Title/Abstract])) OR (SARS Coronavirus 2 Infection[Title/Abstract])) OR (SARS CoV 2 Virus[Title/Abstract])) OR (SARS CoV 2 Infection[Title/Abstract])) OR (SARS-CoV-2 Virus*[Title/Abstract])) OR (SARS-CoV-2 Infection*[Title/Abstract])) OR (Severe Acute Respiratory Syndrome Coronavirus 2[Title/Abstract])) OR (Severe Acute Respiratory Syndrome Coronavirus 2 Infection[Title/Abstract])) OR (Virus Disease, COVID-19[Title/Abstract])) OR (Virus Infection, COVID-19[Title/Abstract])) OR (Virus*, COVID19[Title/Abstract])) OR (Virus, COVID-19[Title/Abstract])) OR (Virus, SARS-CoV-2[Title/Abstract])) OR (Wuhan Coronavirus[Title/Abstract])) OR (Wuhan Seafood Market Pneumonia Virus[Title/Abstract])) OR (postacute sequelae of COVID*[Title/Abstract])) OR (PASC[Title/Abstract])) OR (post-acute sequelae of COVID*[Title/Abstract])) OR (post acute sequelae of COVID*[Title/Abstract])) OR (Long COVID-19[Title/Abstract])) OR (Long-COVID-19[Title/Abstract])) OR (Long-COVID[Title/Abstract])) OR (Long COVID[Title/Abstract])) OR (Long-COVID symptoms[Title/Abstract])) OR (long-term post-COVID[Title/Abstract])) OR (long-term post-COVID symptoms[Title/Abstract])) OR (post-COVID symptoms[Title/Abstract])) OR (long‐term SARS‐Cov‐2 infection[Title/Abstract])) OR (Post-COVID*[Title/Abstract])) OR (Post COVID*[Title/Abstract])) OR (chronic post-coronavirus disease 2019[Title/Abstract])) OR (chronic post-COVID syndrome[Title/Abstract])) OR (chronic post-COVID-19[Title/Abstract])) OR (postacute sequelae of COVID-19[Title/Abstract])) OR (post-COVID-19[Title/Abstract])) OR (post covid-19[Title/Abstract])) AND ((((((((((Excess of weight[Title/Abstract]) OR (Body Mass Index[Title/Abstract])) OR (Obesity[Title/Abstract])) OR (Overweight[Title/Abstract])) OR (Index, Quetelet[Title/Abstract])) OR (Quetelet* Index[Title/Abstract])) OR (Index, Body Mass[Title/Abstract])) OR (Body Mass Index[MeSH Terms])) OR (Obesity[MeSH Terms])) OR (Overweight[MeSH Terms]))) AND (((((((((((((((((((((((((((((((((((((((((((((((((((((((((((((((((((((((((((((((((((((((((((((((((((((((((Symptoms and Signs[MeSH Terms]) OR (Hospitalization*[MeSH Terms])) OR (Patient Discharge*[MeSH Terms])) OR (Patient Readmission*[MeSH Terms])) OR (Fatigue[MeSH Terms])) OR (Dyspnea[MeSH Terms])) OR (Anxiety[MeSH Terms])) OR (Mental Fatigue[MeSH Terms])) OR (Chest Pain[MeSH Terms])) OR (Cognitive Dysfunction[MeSH Terms])) OR (Cough[MeSH Terms])) OR (Depression[MeSH Terms])) OR (Diarrhea[MeSH Terms])) OR (Dizziness[MeSH Terms])) OR (Alopecia[MeSH Terms])) OR (Headache[MeSH Terms])) OR (Arthralgia[MeSH Terms])) OR (Anosmia[MeSH Terms])) OR (Ageusia[MeSH Terms])) OR (Myalgia[MeSH Terms])) OR (Nausea[MeSH Terms])) OR (Pharyngitis[MeSH Terms])) OR (Sputum[MeSH Terms])) OR (Sleep Quality[MeSH Terms])) OR (Symptoms[Title/Abstract] AND Signs[Title/Abstract])) OR (Symptom*[Title/Abstract])) OR (Sequelae[Title/Abstract])) OR (COVID-19 sequelae[Title/Abstract])) OR (Hospitalisation*[Title/Abstract])) OR (Hospitalization*[Title/Abstract])) OR (Hospital admission[Title/Abstract])) OR (Patient Discharge*[Title/Abstract])) OR (Discharge*[Title/Abstract])) OR (Rehospitalization*[Title/Abstract])) OR (Hospital Readmission[Title/Abstract])) OR (Readmission[Title/Abstract])) OR (Patient Readmission*[Title/Abstract])) OR (Effort intolerance[Title/Abstract])) OR (Exercise intolerance[Title/Abstract])) OR (exercise capacity[Title/Abstract])) OR (Walking intolerance[Title/Abstract])) OR (Fatigue[Title/Abstract])) OR (Dyspnea[Title/Abstract])) OR (Shortness of Breath[Title/Abstract])) OR (Breath Shortness[Title/Abstract])) OR (Breathlessness[Title/Abstract])) OR (Angst[Title/Abstract])) OR (Dyspnea[Title/Abstract])) OR (Anxiety[Title/Abstract])) OR (Mental Fatigue[Title/Abstract])) OR (Chest Pain[Title/Abstract])) OR (Angst[Title/Abstract])) OR (Social Anxiet*[Title/Abstract])) OR (Hypervigilance[Title/Abstract])) OR (Nervousness[Title/Abstract])) OR (Anxiousness[Title/Abstract])) OR (Brain Fog[Title/Abstract])) OR (Mental Fog[Title/Abstract])) OR (Chest Pain[Title/Abstract])) OR (Chest Pain*[Title/Abstract])) OR (Palpitation[Title/Abstract])) OR (Cognitive Dysfunction*[Title/Abstract])) OR (Cognitive Impairment*[Title/Abstract])) OR (Cognitive Disorder*[Title/Abstract])) OR (Cognitive Decline*[Title/Abstract])) OR (Mental Deterioration*[Title/Abstract])) OR (Cough*[Title/Abstract])) OR (Depressive Symptom*[Title/Abstract])) OR (Emotional Depression[Title/Abstract])) OR (Diarrhea*[Title/Abstract])) OR (Dizzyness[Title/Abstract])) OR (Lightheadedness[Title/Abstract])) OR (Light-Headedness[Title/Abstract])) OR (Light Headedness[Title/Abstract])) OR (Baldness[Title/Abstract])) OR (Hair Loss*[Title/Abstract])) OR (Headache*[Title/Abstract])) OR (Head Pain*[Title/Abstract])) OR (Cephalalgia*[Title/Abstract])) OR (Cephalgia*[Title/Abstract])) OR (Arthralgia*[Title/Abstract])) OR (Joint Pain*[Title/Abstract])) OR (Loss of Smell[Title/Abstract])) OR (Anosmia[Title/Abstract])) OR (Smell Loss[Title/Abstract])) OR (Hyposmia[Title/Abstract])) OR (Loss of Taste[Title/Abstract])) OR (Taste Loss[Title/Abstract])) OR (Hypogeusia*[Title/Abstract])) OR (Taste-Blindness[Title/Abstract])) OR (Taste Blindness[Title/Abstract])) OR (Ageusia[Title/Abstract])) OR (Myalgia[Title/Abstract])) OR (Muscle Pain[Title/Abstract])) OR (Muscle Soreness*[Title/Abstract])) OR (Muscle Tenderness[Title/Abstract])) OR (Nausea[Title/Abstract])) OR (Pharyngitis[Title/Abstract])) OR (Pharyngitides[Title/Abstract])) OR (Sore Throat*[Title/Abstract])) OR (Sputum*[Title/Abstract])) OR (Sleep Qualit*[Title/Abstract])) OR (Sleep disorder[Title/Abstract])) OR (Sleep difficulty[Title/Abstract])) OR (Smell[Title/Abstract] OR tarte disturbance[Title/Abstract]))** Filters: **Humans** |
| Embase | ('2019 ncov disease':ab,ti OR '2019 ncov infection':ab,ti OR '2019 novel coronavirus disease':ab,ti OR '2019 novel coronavirus infection':ab,ti OR '2019 novel coronavirus*':ab,ti OR '2019-ncov':ab,ti OR '2019-ncov disease*':ab,ti OR '2019-ncov infection*':ab,ti OR 'coronavirus 2, sars':ab,ti OR 'coronavirus disease 19':ab,ti OR 'coronavirus disease 2019':ab,ti OR 'coronavirus disease 2019 virus':ab,ti OR 'coronavirus disease-19':ab,ti OR 'coronavirus, 2019 novel':ab,ti OR 'coronavirus, wuhan':ab,ti OR 'covid 19':ab,ti OR 'covid 19 virus':ab,ti OR 'covid 19 pandemic':ab,ti OR 'covid 19 virus disease':ab,ti OR 'covid 19 virus infection':ab,ti OR 'covid, long-haul':ab,ti OR 'covid19':ab,ti OR 'covid-19 syndrome, post-acute':ab,ti OR 'covid19 virus*':ab,ti OR 'covid-19 pandemic*':ab,ti OR 'covid-19 virus disease*':ab,ti OR 'covid-19 virus infection*':ab,ti OR 'covid-19, long haul':ab,ti OR 'disease 2019, coronavirus':ab,ti OR 'disease, 2019-ncov':ab,ti OR 'disease, covid-19 virus':ab,ti OR 'infection, 2019-ncov':ab,ti OR 'infection, covid-19 virus':ab,ti OR 'infection, sars-cov-2':ab,ti OR 'long haul covid 19*':ab,ti OR 'long haul covid*':ab,ti OR 'long haul covid-19':ab,ti OR 'long-haul covid':ab,ti OR 'novel coronavirus, 2019':ab,ti OR 'pandemic, covid-19':ab,ti OR 'post acute covid-19 syndrome*':ab,ti OR 'post acute sequelae of sars cov 2 infection':ab,ti OR 'post covid conditions':ab,ti OR 'post-acute sequelae of sars-cov-2 infection':ab,ti OR 'post-covid condition*':ab,ti OR 'sars coronavirus 2':ab,ti OR 'sars coronavirus 2 infection':ab,ti OR 'sars cov 2 virus':ab,ti OR 'sars cov 2 infection':ab,ti OR 'sars-cov-2 virus*':ab,ti OR 'sars-cov-2 infection*':ab,ti OR 'severe acute respiratory syndrome coronavirus 2':ab,ti OR 'severe acute respiratory syndrome coronavirus 2 infection':ab,ti OR 'virus disease, covid-19':ab,ti OR 'virus infection, covid-19':ab,ti OR 'virus*, covid19':ab,ti OR 'virus, covid-19':ab,ti OR 'virus, sars-cov-2':ab,ti OR 'wuhan coronavirus':ab,ti OR 'wuhan seafood market pneumonia virus':ab,ti OR 'post acute covid 19 syndrome':ab,ti OR 'covid-19':ab,ti OR 'sars-cov-2':ab,ti OR 'postacute sequelae of covid*':ab,ti OR 'pasc':ab,ti OR 'post-acute sequelae of covid*':ab,ti OR 'post acute sequelae of covid*':ab,ti OR 'long covid-19':ab,ti OR 'long-covid-19':ab,ti OR 'long-covid':ab,ti OR 'long covid':ab,ti OR 'long-covid symptoms':ab,ti OR 'long-term post-covid':ab,ti OR 'long-term post-covid symptoms':ab,ti OR 'post-covid symptoms':ab,ti OR 'long‐term sars‐cov‐2 infection':ab,ti OR 'post-covid*':ab,ti OR 'post covid*':ab,ti OR 'chronic post-coronavirus disease 2019':ab,ti OR 'chronic post-covid syndrome':ab,ti OR 'chronic post-covid-19':ab,ti) AND ('excess of weight':ab,ti OR overweight:ab,ti OR obesity:ab,ti OR 'body mass index':ab,ti OR 'index, body mass':ab,ti OR 'quetelet* index':ab,ti OR 'index, quetelet':ab,ti) AND ('symptoms and signs':ab,ti OR 'hospitalization*':ab,ti OR 'patient discharge*':ab,ti OR 'patient readmission*':ab,ti OR fatigue:ab,ti OR dyspnea:ab,ti OR anxiety:ab,ti OR 'mental fatigue':ab,ti OR 'chest pain':ab,ti OR 'cognitive dysfunction':ab,ti OR cough:ab,ti OR depression:ab,ti OR diarrhea:ab,ti OR dizziness:ab,ti OR alopecia:ab,ti OR headache:ab,ti OR arthralgia:ab,ti OR anosmia:ab,ti OR ageusia:ab,ti OR myalgia:ab,ti OR nausea:ab,ti OR pharyngitis:ab,ti OR sputum:ab,ti OR 'sleep quality':ab,ti OR symptom*:ab,ti OR sequelae:ab,ti OR 'covid-19 sequelae':ab,ti OR hospitalisation*:ab,ti OR 'hospital admission':ab,ti OR 'patient discharge':ab,ti OR discharge*:ab,ti OR rehospitalization*:ab,ti OR 'hospital readmission':ab,ti OR readmission:ab,ti OR 'effort intolerance':ab,ti OR 'exercise intolerance':ab,ti OR 'exercise capacity':ab,ti OR 'walking intolerance':ab,ti OR 'shortness of breath':ab,ti OR 'breath shortness':ab,ti OR breathlessness:ab,ti OR angst:ab,ti OR 'social anxiet':ab,ti OR hypervigilance:ab,ti OR nervousness:ab,ti OR anxiousness:ab,ti OR 'brain fog':ab,ti OR 'mental fog':ab,ti OR palpitation:ab,ti OR 'cognitive impairment*':ab,ti OR 'cognitive disorder*':ab,ti OR 'cognitive decline*':ab,ti OR 'mental deterioration*':ab,ti OR 'depressive symptom*':ab,ti OR 'emotional depression':ab,ti OR lightheadedness:ab,ti OR 'light-headedness':ab,ti OR 'light headedness':ab,ti OR baldness:ab,ti OR 'hair loss*':ab,ti OR 'head pain*':ab,ti OR cephalalgia*:ab,ti OR cephalgia*:ab,ti OR 'joint pain*':ab,ti OR 'loss of smell':ab,ti OR 'smell loss':ab,ti OR hyposmia:ab,ti OR 'loss of taste':ab,ti OR 'taste loss':ab,ti OR hypogeusia*:ab,ti OR 'taste-blindness':ab,ti OR 'taste blindness':ab,ti OR 'muscle pain':ab,ti OR 'muscle soreness*':ab,ti OR 'muscle tenderness':ab,ti OR pharyngitides:ab,ti OR 'sore throat*':ab,ti OR 'sleep qualit*':ab,ti OR 'sleep disorder':ab,ti OR 'sleep difficulty':ab,ti OR 'smell or tarte disturbance':ab,ti OR 'difficulty concentration':ab,ti) |
| BVS | ((post acute covid 19 syndrome) OR (covid-19) OR (sars-cov-2) OR (long covid-19) OR (2019 ncov disease) OR (2019 ncov infection) OR (2019 novel coronavirus disease) OR (2019 novel coronavirus infection) OR (2019 novel coronavirus*) OR (2019-ncov) OR (2019-ncov disease*) OR (2019-ncov infection*) OR (coronavirus 2, sars) OR (coronavirus disease 19) OR (coronavirus disease 2019) OR (coronavirus disease 2019 virus) OR (coronavirus disease-19) OR (coronavirus, 2019 novel) OR (coronavirus, wuhan) OR (covid 19) OR (covid 19 virus) OR (covid 19 pandemic) OR (covid 19 virus disease) OR (covid 19 virus infection) OR (covid, long-haul) OR (covid19) OR (covid-19 syndrome, post-acute) OR (covid19 virus*) OR (covid-19 pandemic*) OR (covid-19 virus disease*) OR (covid-19 virus infection*) OR (covid-19, long haul) OR (disease 2019, coronavirus) OR (disease, 2019-ncov) OR (disease, covid-19 virus) OR (infection, 2019-ncov) OR (infection, covid-19 virus) OR (infection, sars-cov-2) OR (long covid) OR (long haul covid 19*) OR (long haul covid*) OR (long haul covid-19) OR (long-haul covid) OR (novel coronavirus, 2019) OR (pandemic, covid-19) OR (post acute covid 19 syndrome) OR (post acute covid-19 syndrome*) OR (post acute sequelae of sars cov 2 infection) OR (post covid conditions) OR (post-acute sequelae of sars-cov-2 infection) OR (post-covid condition*) OR (sars coronavirus 2) OR (sars coronavirus 2 infection) OR (sars cov 2 virus) OR (sars cov 2 infection) OR (sars-cov-2 virus*) OR (sars-cov-2 infection*) OR (severe acute respiratory syndrome coronavirus 2) OR (severe acute respiratory syndrome coronavirus 2 infection) OR (virus disease, covid-19) OR (virus infection, covid-19) OR (virus*, covid19) OR (virus, covid-19) OR (virus, sars-cov-2) OR (wuhan coronavirus) OR (wuhan seafood market pneumonia virus) OR (postacute sequelae of covid*) OR (pasc) OR (post-acute sequelae of covid*) OR (post acute sequelae of covid*) OR (long covid-19) OR (long-covid-19) OR (long-covid) OR (long covid) OR (long-covid symptoms) OR (long-term post-covid) OR (long-term post-covid symptoms) OR (post-covid symptoms) OR (long‐term sars‐cov‐2 infection) OR (post-covid*) OR (post covid*) OR (chronic post-coronavirus disease 2019) OR (chronic post-covid syndrome) OR (chronic post-covid-19) OR (postacute sequelae of covid-19) OR (post-covid-19) OR (post covid-19)) AND ((excess of weight) OR (body mass index) OR (obesity) OR (overweight) OR (index, quetelet) OR (quetelet* index) OR (index, body mass)) AND ((symptoms AND signs) OR (symptom*) OR (sequelae) OR (signs AND symptoms) OR (hospitalisation*) OR (hospitalization*) OR (hospital admission) OR (discharge*) OR (patient discharge*) OR (patient readmission) OR (hospital readmission)  OR (readmission*) OR (rehospitalization*) OR (fatigue) OR (effort intolerance) OR (exercise intolerance) OR (exercise capacity) OR (walking intolerance) OR (shortness of breath) OR (breath shortness) OR (breathlessness) OR (dyspnea) OR (angst) OR (social anxiet*) OR (hypervigilance) OR (nervousness) OR (anxiousness) OR (anxiety) OR (difficulty concentration) OR (brain fog) OR (mental fog) OR (mental fatigue) OR (chest pain*) OR (palpitation) OR (cognitive dysfunction*) OR (cognitive impairment*) OR (cognitive disorder*) OR (cognitive decline*) OR (mental deterioration*) OR (cough*) OR (depressive symptom*) OR (emotional depression) OR (depression*) OR (diarrhea*) OR (baldness) OR (hair loss*) OR (alopecia) OR (headache*) OR (head pain*) OR (cephalalgia*) OR (cephalgia*) OR (arthralgia*) OR (joint pain*) OR (anosmia) OR (loss of smell) OR (smell loss) OR (hyposmia) OR (ageusia) OR (loss of taste) OR (taste loss) OR (hypogeusia*) OR (taste-blindness) OR (taste blindness) OR (smell OR tarte disturbance) OR (myalgia) OR (muscle pain) OR (muscle soreness*) OR (muscle tenderness) OR (nausea) OR (pharyngitides) OR (pharyngitis) OR (sore throat*) OR (sputum*) OR (sleep qualit*) OR (sleep disorder) OR (sleep difficulty)) AND ( limit:("humans")) |
| SCOPUS | ( ( TITLE-ABS-KEY ( "SARS Coronavirus 2 Infection" OR "SARS CoV 2 Virus" OR "SARS CoV 2 Infection" OR "SARS-CoV-2 Virus*" OR "SARS-CoV-2 Infection*" OR "Severe Acute Respiratory Syndrome Coronavirus 2" OR "Severe Acute Respiratory Syndrome Coronavirus 2 Infection" OR "Virus Disease, COVID-19" OR "Virus Infection, COVID-19" OR "Virus*, COVID19" OR "Virus, COVID-19" OR "Virus, SARS-CoV-2" OR "Wuhan Coronavirus" OR "Wuhan Seafood Market Pneumonia Virus" OR "postacute sequelae of COVID*" OR "PASC" OR "post-acute sequelae of COVID*" OR "post acute sequelae of COVID*" OR "Long COVID-19" OR "Long-COVID-19" OR "Long-COVID" OR "Long COVID" OR "Long-COVID symptoms" OR "long-term post-COVID" OR "long-term post-COVID symptoms" OR "post-COVID symptoms" OR "long‐term SARS‐Cov‐2 infection" OR "Post-COVID*" OR "Post COVID*" OR "chronic post-coronavirus disease 2019" OR "chronic post-COVID syndrome" OR "chronic post-COVID-19" ) ) OR ( TITLE-ABS-KEY ( "Infection, SARS-CoV-2" OR "Long COVID" OR "Long Haul COVID 19*" OR "Long Haul COVID*" OR "Long Haul COVID-19" OR "Long-Haul COVID" OR "Novel Coronavirus, 2019" OR "Pandemic, COVID-19" OR "Post Acute COVID 19 Syndrome" OR "Post Acute COVID-19 Syndrome*" OR "Post Acute Sequelae of SARS CoV 2 Infection" OR "Post COVID Conditions" OR "Post-Acute Sequelae of SARS-CoV-2 Infection" OR "Post-COVID Condition*" OR "SARS Coronavirus 2" ) ) OR ( TITLE-ABS-KEY ( "Long COVID-19" OR "Post acute covid 19 syndrome" OR "COVID-19" OR "SARS-CoV-2" OR "2019 nCoV Disease" OR "2019 nCoV Infection" OR "2019 Novel Coronavirus Disease" OR "2019 Novel Coronavirus Infection" OR "2019 Novel Coronavirus*" OR "2019-nCoV" OR "2019-nCoV Disease*" OR "2019-nCoV Infection*" OR "Coronavirus 2, SARS" OR "Coronavirus Disease 19" OR "Coronavirus Disease 2019" OR "Coronavirus Disease 2019 Virus" OR "Coronavirus Disease-19" OR "Coronavirus, 2019 Novel" OR "Coronavirus, Wuhan" OR "COVID 19" OR "COVID 19 Virus" OR "COVID 19 Pandemic" OR "COVID 19 Virus Disease" OR "COVID 19 Virus Infection" OR "COVID, Long-Haul" OR "COVID19" OR "COVID-19 Syndrome, Post-Acute" OR "COVID19 Virus*" OR "COVID-19 Pandemic*" OR "COVID-19 Virus Disease*" OR "COVID-19 Virus Infection*" OR "COVID-19, Long Haul" OR "Disease 2019, Coronavirus" OR "Disease, 2019-nCoV" OR "Disease, COVID-19 Virus" OR "Infection, 2019-nCoV" OR "Infection, COVID-19 Virus" ) ) ) AND ( TITLE-ABS-KEY ( "Excess of weight" OR overweight OR obesity OR "Body Mass Index" OR "Index, Body Mass" OR "Quetelet* Index" OR "Index, Quetelet" ) ) AND ( TITLE-ABS-KEY ( "Symptoms and Signs" OR "Hospitalization*" OR "Patient Discharge*" OR "Patient Readmission*" OR fatigue OR dyspnea OR anxiety OR "Mental Fatigue" OR "Chest Pain" OR "Cognitive Dysfunction" OR cough OR depression OR diarrhea OR dizziness OR alopecia OR headache OR arthralgia OR anosmia OR ageusia OR myalgia OR nausea OR pharyngitis OR sputum OR "Sleep Quality" OR symptom* OR sequelae OR "COVID-19 sequelae" OR hospitalisation* OR "Hospital admission" OR "Patient Discharge" OR discharge* OR rehospitalization* OR "Hospital Readmission" OR readmission OR "Effort intolerance" OR "Exercise intolerance" OR "exercise capacity" OR "Walking intolerance" OR "Shortness of Breath" OR "Breath Shortness" OR breathlessness OR angst OR "Social Anxiet" OR hypervigilance OR nervousness OR anxiousness OR "Brain Fog" OR "Mental Fog" OR palpitation OR "Cognitive Impairment*" OR "Cognitive Disorder*" OR "Cognitive Decline*" OR "Mental Deterioration*" OR "Depressive Symptom*" OR "Emotional Depression" OR lightheadedness OR "Light-Headedness" OR "Light Headedness" OR baldness OR "Hair Loss*" OR "Head Pain*" OR cephalalgia* OR cephalgia* OR "Joint Pain*" OR "Loss of Smell" OR "Smell Loss" OR hyposmia OR "Loss of Taste" OR "Taste Loss" OR hypogeusia* OR "Taste-Blindness" OR "Taste Blindness" OR "Muscle Pain" OR "Muscle Soreness*" OR "Muscle Tenderness" OR pharyngitides OR "Sore Throat*" OR "Sleep Qualit*" OR "Sleep disorder" OR "Sleep difficulty" OR "Smell OR tarte disturbance" OR "Difficulty concentration" ) ) AND ( LIMIT-TO ( EXACTKEYWORD , "Human" ) ) |
| WEB OF SCIENCE | ((AB=(Long COVID-19 OR Post acute covid 19 syndrome OR COVID-19 OR SARS-CoV-2 OR 2019 nCoV Disease OR 2019 nCoV Infection OR 2019 Novel Coronavirus Disease OR 2019 Novel Coronavirus Infection OR 2019 Novel Coronavirus* OR 2019-nCoV OR 2019-nCoV Disease* OR 2019-nCoV Infection* OR Coronavirus 2, SARS OR Coronavirus Disease 19 OR Coronavirus Disease 2019 OR Coronavirus Disease 2019 Virus OR Coronavirus Disease-19 OR Coronavirus, 2019 Novel OR Coronavirus, Wuhan OR COVID 19 OR COVID 19 Virus OR COVID 19 Pandemic OR COVID 19 Virus Disease OR COVID 19 Virus Infection OR COVID, Long-Haul OR COVID19 OR COVID-19 Syndrome, Post-Acute OR COVID19 Virus* OR COVID-19 Pandemic* OR COVID-19 Virus Disease* OR COVID-19 Virus Infection* OR COVID-19, Long Haul OR Disease 2019, Coronavirus OR Disease, 2019-nCoV OR Disease, COVID-19 Virus OR Infection, 2019-nCoV OR Infection, COVID-19 Virus OR Infection, SARS-CoV-2 OR Long COVID OR Long Haul COVID 19* OR Long Haul COVID* OR Long Haul COVID-19 OR Long-Haul COVID OR Novel Coronavirus, 2019 OR Pandemic, COVID-19 OR Post Acute COVID 19 Syndrome OR Post Acute COVID-19 Syndrome* OR Post Acute Sequelae of SARS CoV 2 Infection OR Post COVID Conditions OR Post-Acute Sequelae of SARS-CoV-2 Infection OR Post-COVID Condition* OR SARS Coronavirus 2 OR SARS Coronavirus 2 Infection OR SARS CoV 2 Virus OR SARS CoV 2 Infection OR SARS-CoV-2 Virus* OR SARS-CoV-2 Infection* OR Severe Acute Respiratory Syndrome Coronavirus 2 OR Severe Acute Respiratory Syndrome Coronavirus 2 Infection OR Virus Disease, COVID-19 OR Virus Infection, COVID-19 OR Virus*, COVID19 OR Virus, COVID-19 OR Virus, SARS-CoV-2 OR Wuhan Coronavirus OR Wuhan Seafood Market Pneumonia Virus OR postacute sequelae of COVID* OR PASC OR post-acute sequelae of COVID* OR post acute sequelae of COVID* OR Long COVID-19 OR Long-COVID-19 OR Long-COVID OR Long COVID OR Long-COVID symptoms OR long-term post-COVID OR long-term post-COVID symptoms OR post-COVID symptoms OR long‐term SARS‐Cov‐2 infection OR Post-COVID* OR Post COVID* OR chronic post-coronavirus disease 2019 OR chronic post-COVID syndrome OR chronic post-COVID-19)) AND AB=(Excess of weight OR Overweight OR Obesity OR Body Mass Index OR Index, Body Mass OR Quetelet* Index OR Index, Quetelet)) AND AB=(Symptoms and Signs OR Hospitalization* OR Patient Discharge* OR Patient Readmission* OR Fatigue OR Dyspnea OR Anxiety OR Mental Fatigue OR Chest Pain OR Cognitive Dysfunction OR Cough OR Depression OR Diarrhea OR Dizziness OR Alopecia OR Headache OR Arthralgia OR Anosmia OR Ageusia OR Myalgia OR Nausea OR Pharyngitis OR Sputum OR Sleep Quality OR Symptom* OR Sequelae OR COVID-19 sequelae OR Hospitalisation* OR Hospital admission OR Patient Discharge OR Discharge* OR Rehospitalization* OR Hospital Readmission OR Readmission OR Effort intolerance OR Exercise intolerance OR exercise capacity OR Walking intolerance OR Shortness of Breath OR Breath Shortness OR Breathlessness OR Angst OR Social Anxiet OR Hypervigilance OR Nervousness OR Anxiousness OR Brain Fog OR Mental Fog OR Palpitation OR Cognitive Impairment* OR Cognitive Disorder* OR Cognitive Decline* OR Mental Deterioration* OR Depressive Symptom* OR Emotional Depression OR Lightheadedness OR Light-Headedness OR Light Headedness OR Baldness OR Hair Loss* OR Head Pain* OR Cephalalgia* OR Cephalgia* OR Joint Pain* OR Loss of Smell OR Smell Loss OR Hyposmia OR Loss of Taste OR Taste Loss OR Hypogeusia* OR Taste-Blindness OR Taste Blindness OR Muscle Pain OR Muscle Soreness* OR Muscle Tenderness OR Pharyngitides OR Sore Throat* OR Sleep Qualit* OR Sleep disorder OR Sleep difficulty OR Smell OR tarte disturbance OR Difficulty concentration) |
| PROQUEST | AB,TI("Post acute covid 19 syndrome" OR "COVID-19" OR "SARS-CoV-2" OR "Long COVID-19" OR "2019 nCoV Disease" OR "2019 nCoV Infection" OR "2019 Novel Coronavirus Disease" OR "2019 Novel Coronavirus Infection" OR "2019 Novel Coronavirus*" OR "2019-nCoV" OR "2019-nCoV Disease*" OR "2019-nCoV Infection*" OR "Coronavirus 2, SARS" OR "Coronavirus Disease 19" OR "Coronavirus Disease 2019" OR "Coronavirus Disease 2019 Virus" OR "Coronavirus Disease-19" OR "Coronavirus, 2019 Novel" OR "Coronavirus, Wuhan" OR "COVID 19" OR "COVID 19 Virus" OR "COVID 19 Pandemic" OR "COVID 19 Virus Disease" OR "COVID 19 Virus Infection" OR "COVID, Long-Haul" OR "COVID19" OR "COVID-19 Syndrome, Post-Acute" OR "COVID19 Virus*" OR "COVID-19 Pandemic*" OR "COVID-19 Virus Disease*" OR "COVID-19 Virus Infection*" OR "COVID-19, Long Haul" OR "Disease 2019, Coronavirus" OR "Disease, 2019-nCoV" OR "Disease, COVID-19 Virus" OR "Infection, 2019-nCoV" OR "Infection, COVID-19 Virus" OR "Infection, SARS-CoV-2" OR "Long COVID" OR "Long Haul COVID 19*" OR "Long Haul COVID*" OR "Long Haul COVID-19" OR "Long-Haul COVID" OR "Novel Coronavirus, 2019" OR "Pandemic, COVID-19" OR "Post Acute COVID 19 Syndrome" OR "Post Acute COVID-19 Syndrome*" OR "Post Acute Sequelae of SARS CoV 2 Infection" OR "Post COVID Conditions" OR "Post-Acute Sequelae of SARS-CoV-2 Infection" OR "Post-COVID Condition*" OR "SARS Coronavirus 2" OR "SARS Coronavirus 2 Infection" OR "SARS CoV 2 Virus" OR "SARS CoV 2 Infection" OR "SARS-CoV-2 Virus*" OR "SARS-CoV-2 Infection*" OR "Severe Acute Respiratory Syndrome Coronavirus 2" OR "Severe Acute Respiratory Syndrome Coronavirus 2 Infection" OR "Virus Disease, COVID-19" OR "Virus Infection, COVID-19" OR "Virus*, COVID19" OR "Virus, COVID-19" OR "Virus, SARS-CoV-2" OR "Wuhan Coronavirus" OR "Wuhan Seafood Market Pneumonia Virus" OR "postacute sequelae of COVID*"  OR "PASC" OR "post-acute sequelae of COVID*" OR "post acute sequelae of COVID*" OR "Long COVID-19" OR "Long-COVID-19" OR "Long-COVID" OR "Long COVID" OR "Long-COVID symptoms" OR "long-term post-COVID" OR "long-term post-COVID symptoms" OR "post-COVID symptoms" OR "long‐term SARS‐Cov‐2 infection" OR "Post-COVID*" OR "Post COVID*" OR "chronic post-coronavirus disease 2019" OR "chronic post-COVID syndrome" OR "chronic post-COVID-19" OR "postacute sequelae of COVID-19" OR "post-COVID-19" OR "post covid-19") AND AB,TI("Excess of weight" OR "Body Mass Index" OR "Obesity" OR "Overweight" OR "Index, Quetelet" OR "Quetelet* Index" OR "Index, Body Mass") AND AB,TI("Symptoms and Signs" OR "Symptom*" OR "sequelae" OR "Signs and Symptoms" OR "Hospitalisation*" OR "Hospitalization*" OR "Hospital admission" OR "Discharge*" OR "Patient Discharge*" OR "Patient readmission" OR "Hospital readmission"  OR "Readmission*" OR "Rehospitalization*" OR "Fatigue" OR "Effort intolerance" OR "Exercise intolerance" OR "exercise capacity" OR "Walking intolerance" OR "Shortness of Breath" OR "Breath Shortness" OR "Breathlessness" OR "Dyspnea" OR "Angst" OR "Social Anxiet*" OR "Hypervigilance" OR "Nervousness" OR "Anxiousness" OR "Anxiety" OR "Difficulty concentration" OR "Brain Fog" OR "Mental Fog" OR "Mental Fatigue" OR "Chest Pain*" OR "Palpitation" OR "Cognitive Dysfunction*" OR "Cognitive Impairment*" OR "Cognitive Disorder*" OR "Cognitive Decline*" OR "Mental Deterioration*" OR "Cough*" OR "Depressive Symptom*" OR "Emotional Depression" OR "Depression*" OR "Diarrhea*" OR "Baldness" OR "Hair Loss*" OR "Alopecia" OR "Headache*" OR "Head Pain*" OR "Cephalalgia*" OR "Cephalgia*" OR "Arthralgia*" OR "Joint Pain*" OR "Anosmia" OR "Loss of Smell" OR "Smell Loss" OR "Hyposmia" OR "Ageusia" OR "Loss of Taste" OR "Taste Loss" OR "Hypogeusia*" OR "Taste-Blindness" OR "Taste Blindness" OR "Smell or tarte disturbance" OR "Myalgia" OR "Muscle Pain" OR "Muscle Soreness*" OR "Muscle Tenderness" OR "Nausea" OR "Pharyngitides" OR "Pharyngitis" OR "Sore Throat*" OR "Sputum*" OR "Sleep Qualit*" OR "Sleep disorder" OR "Sleep difficulty") |
| MedXirv | ""Post acute covid 19 syndrome" OR "Post-COVID Condition*" AND "Obesity" OR "Overweight"" (match all words) |
| Google Schoolar | (“Long COVID*” OR “Post acute covid 19 syndrome” OR “COVID-19” OR “SARS-CoV-2” OR  “COVID 19” OR   “COVID 19 Virus Disease” OR “COVID19” OR “COVID19 Virus*” OR “COVID-19 Pandemic*” OR “Long COVID” OR “Long Haul COVID*” OR “Post Acute COVID 19 Syndrome” OR “Post Acute COVID-19 Syndrome*” OR “Post Acute Sequelae of SARS CoV 2 Infection” OR  “Post COVID Conditions” OR “Post-Acute Sequelae of SARS-CoV-2 Infection” OR “Post-COVID Condition*” OR “postacute sequelae of COVID*” OR “PASC” OR  “post-acute sequelae of COVID*” OR “post acute sequelae of COVID*” OR  “Long-COVID symptoms” OR  “long-term post-COVID” OR  “long-term post-COVID symptoms” OR “post-COVID symptoms” OR “long‐term SARS‐Cov‐2 infection” OR “Post-COVID*” OR “Post COVID*” OR “chronic post-coronavirus disease 2019” OR “chronic post-COVID syndrome” OR “chronic post-COVID-19”) AND (“Excess of weight” OR Overweight OR Obesity OR “Body Mass Index” OR  “Quetelet* Index”) AND (“Symptoms and Signs” OR “Hospitalization*” OR “Patient Discharge*” OR “Patient Readmission*” OR Fatigue OR Dyspnea OR Anxiety OR “Mental Fatigue” OR “Chest Pain” OR “Cognitive Dysfunction” OR Cough OR Depression OR Diarrhea OR Dizziness OR Alopecia OR Headache OR Arthralgia OR Anosmia OR Ageusia OR Myalgia OR Nausea OR Pharyngitis OR Sputum OR “Sleep Qualit*” OR Symptom* OR Sequelae OR “COVID-19 sequelae” OR “Hospital admission” OR Discharge* OR Rehospitalization* OR Readmission OR “Exercise intolerance” OR “exercise capacity” OR “Walking intolerance” OR “Shortness of Breath” OR “Breath Shortness” OR Breathlessness OR Angst  OR OR Hypervigilance OR Nervousness OR Anxiousness OR “Brain Fog” OR “Mental Fog” OR Palpitation OR “Cognitive Impairment*” OR “Cognitive Disorder*” OR “Cognitive Decline*” OR “Depressive Symptom*” OR Baldness OR “Hair Loss*” OR “Head Pain*” OR “Joint Pain*” OR “Loss of Smell” OR “Smell Loss” OR Hyposmia OR “Loss of Taste” OR “Taste Loss” OR Hypogeusia*  OR “Muscle Pain” OR “Sore Throat*” OR “Sleep disorder” OR “Sleep difficulty” OR “Difficulty concentration”) |
