# Supplementary material for: Excess weight is associated with neurological and neuropsychiatric symptoms in post-COVID-19 condition: A systematic review and meta-analysis
Source: PLoS One. 2025 May 7;20(5):e0314892. doi: 10.1371/journal.pone.0314892 (PMC12057935; doi:10.1371/journal.pone.0314892)
Supplement: S3 Table — a All data was extracted by DBR and LOM; b Due to the number of columns the table was split in two to enable the presentation of all data extracted; PCR: polymerase chain reaction; NA: Data non-available. (DOCX) [file pone.0314892.s003.docx]

**Supporting Information**

**S3 Table: Data extracted from included studies (n=18)^a,b^.**

|  | **Author, year** | **Country** | **Aim of the study** | **Study design** | **Specific population (hospitalized, outpatient or mixed)** | **Population criteria evaluation method** | **Data collection instrument** |
| --- | --- | --- | --- | --- | --- | --- | --- |
| 1 | Alkwai, H.M. *et al.,* 2022 [44] | Saudi Arabia | To analyse the persistence of COVID-19 symptoms beyond 3 months or more of disease onset and return to the usual state of health in a mixed population of COVID-19 survivors. | Cross-sectional study | Users of social media platforms with a self-reported diagnosis of COVID-19 based on the assessment of a healthcare worker or a positive COVID-19 test. | Self-reported diagnosis of COVID-19 based on the assessment of a healthcare worker or a positive COVID-19 test. | Ad hoc web-based survey (online survey) |
| 2 | Blümel, J.E. *et al.*, 2022 [53] | 9 Latin-American countries: Argentina, Brazil, Chile, Dominican Republic, Ecuador, Mexico, Panama, Paraguay and Peru | To study the symptoms that appear after the acute-phase of COVID-19 in middle-aged women and delving into the impact this disease has on their mental health and quality of life | Cross-sectional study (multi-national investigation) | Women aged 40-64 who attended a routine health checkup in private centres | Woman diagnosed as PT-PCR positive for SARS-CoV-2 | Interviews during general genecology consultation |
| 3 | Bungenberg, J. *et al*.,  2022 [48] | Germany | To objectify and compare persisting self-reported symptoms in initially hospitalized and non-hospitalized patients after infection | Cross-sectional | (Mixed population) the first 50 included patients, grouped in initially hospitalized (n = 21) and non-hospitalized (n = 29) patients during acute COVID-19 at the Department of Neurology, RWTH Aachen University Hospital in Germany | COVID-19 confirmed by reverse transcription polymerase chain reaction of nasopharyngeal swab or the presence of SARS-CoV-2-antibodies without previous vaccination. | A structured interview was performed by an attending neurologist to identify current and past acute symptoms |
| 4 | Carter, S.J. *et al.*, 2022 [54] | USA | To compared functional status, mood state, and leisure-time PA in women with and without a history of SARS-CoV-2 matched for age and body mass index (BMI). | Case-control | Woman of community living close by the university of Indiana | Positive laboratory test for SARS-CoV-2 | Interview (presential); A modified Pulmonary Functional Status and Dyspnoea Questionnaire (PFSDQ-M) and The Profile of Mood States (POMS) was used to assess both specific and general mood. |
| 5 | Chudzik, M. *et al.*, 2022 [50] | Poland | To analyse the prevalence of self-reported smell and/or taste disorders among a group of convalescent patients and to identify risk factors for the disease. | Cohort study | Mixed: patients who received hospital treatment and those who received outpatient treatment | Information about patients visiting healthcare centres because of persistent clinical symptoms after SARS-CoV-2 infection recovery - smell and taste disorders (medical registry) | 1. initial visit "0" - health questionnaires and physical examination (sociodemographic information, comorbidities, covid-19 infection severity); 2. health questionnaires at 3 months (symptoms post covid) |
| 6 | Desgranges, F. *et al*., 2022 [52] | Switzerland | Compare the prevalence of symptoms persistent for more than 3 months between COVID-positive and COVID-negative patients in outpatients clinics, and to identify predictors of persistent symptoms in COVID-positive. | Cohort study | Outpatient | SARS-CoV-2 real‐time reverse transcription‐polymerase chain reaction (RT‐PCR) test | 1) Electronical medical records (demographics, medical history, initial clinical presentation and SARS-CoV-2 (RT-PCR) cycle thresholds. 2) structured and standardized phone survey (persistent symptoms at the 7time of the call; secondary hospital admission; medical consultation for persistent symptoms and anthropometric data) |
| 7 | Epsi, N.J. *et al.*, 2024 [55] | United states | Improve the definition of PCC with a data-driven approach to phenotyping. | Cohort | Beneficiaries of U.S Military Health System | Positive polymerase chain reaction (PCR) on a swab collected in the clinic | Medical records or self-report |
| 8 | Farhanah, N. *et al*., 2022 [42] | Indonesia | To determine persistent symptoms and evaluate QoL of COVID-19 patients 3 months after discharge from Dr. Kariadi Hospital Semarang. | Cohort study | Covid-19 patients discharged from Kariadi Hospital Semarang | Covid-19 patients | 1) Electronic medical records (hospital admission data, demographics, clinical signs and symptoms of covid-19); 2) telephone interviews (persistent symptoms, HRQoL questionnaire) |
| 9 | Fernández-de- Las-  Peñas, C. *et al, 2021* [51] | Spain | To investigate a possible association of obesity with long‐term post‐COVID symptoms in hospitalised COVID‐19 survivors. | Case-control | Hospitalised individuals | Hospitalised individuals with a positive diagnosis of COVID‐19 by a real‐time reverse transcription‐polymerase chain reaction (RT‐PCR) and also consistent radiological findings | Medical records (clinical and hospitalisation data); telephonic interview by trained healthcare professionals (long-COVID-19 symptoms). |
| *10* | Gaur, R. *et al*., 2022 [41] | India | Assess the extent of disability following COVID-19 infection using World Health Organization Disability Assessment Schedule 2.0 (WHODAS 2.0) | Cross-sectional study | Patients recovered after COVID-19 infection (hospitalized and home quarantined) Out-Patient Department of Physical Medicine and Rehabilitation patients | All patients who recovered after COVID-19 infection [initial infection confirmed by real time polymerase chain reaction] | Interviews (demographic, pre-existing comorbidity and symptom profile during COVID-19 infection, WHODAS 2.0). |
| *11* | Li, Z. *et al*., 2023^d^ [32] | China | To investigate the mental health status of COVID-19 survivors 1 year after discharge from hospital and reveal the related risk factors. | Cross-sectional | Hospitalized | COVID-19 diagnosis based on Chinese standart | Interview (presential); use of questionnaires and scales 7-Items Generalized Anxiety Disorder Scale (GAD-7) was used to screen the anxiety. The Patient Health Questionnaire-9 (PHQ-9) was used to assess depression status. The Pittsburgh Sleep Quality Index (PSQI) was used to estimate sleep quality |
| 12 | Miyazato, Y. *et al.,* 2022 [43] | Japan | To explore the factors involved in the development and persistence of post-COVID conditions in a cohort of patients recovering from COVID-19 at a hospital in Japan | Cross-sectional | Participants who had recovered from COVID-19 and who visited the outpatient service of the Disease Control and Prevention Center, from Feb 2020 to march 2021 to undergo a pre-donation screening test for COVID-19 convalescent plasmapheresis | Positive for screening test for COVID-19 | Self-report questionnaire by e-mail (patient characteristics , information of acute phase of COVID-19, presence and duration of symptoms related to COVID-19 |
| 13 | Moy, F.M. *et al.*, 2022 [40] | Malaysia | To investigate the mental health status in the form of depression among COVID-19 survivors in the community and its associated factors | Cross-sectional study | Covid-19 survivors | All COVID-19 self reported cases were confirmed via reverse transcription  polymerase chain reaction (RT-PCR) tests. | Online questionnaire - REDCap |
| 14 | Shang, L. *et al.*, 2021^d^ [33] | China | Determine whether obesity has a long‐term impact on COVID‐19 recovery | Cohort study | Patients admitted to and discharged from Wuhan Union Hospital | Laboratory‐confirmed COVID-19 patients who were discharged from Wuhan Union Hospital between | Physical examination, questionnaire surveys during appointment |
| 15 | Sørensen, A.I.V, *et al*., 2022 [45] | Denmark | To estimate the risk difference between COVID-19 positive and negative individuals; to evaluate the duration of symptoms; to explore the influence of risk factors on persistent symptoms | Cross-sectional | Outpatient | PCR | Online survey and electronic records |
| 16 | Van-Wijhe, M. *et al.,* 2022 [46] | Denmark | To investigate the occurrence and risk factors for long-COVID symptoms and health-related quality of life among PCR-confirmed nonhospitalized respondents | Cross-sectional | Mixed (96,8% non-hospitalized and 3,21% hospitalized) - Belgian defence personnel | Positive PCR test | Online survey / questionnaire |
| 17 | Vassalini, P. *et al.*, 2021 [49] | Italy | To assess the prevalence of depressive symptoms and related risk factors at 3 months after discharge to home care following hospitalization for COVID-19 infection. | Cross-sectional | Participants were admitted to the Division of Infectious Diseases, at the Department of Public Health and Infectious Diseases of Umberto I “Sapienza” University Hospital of Rome. | Confirmed case of COVID-19 | 1. Clinical records (clinical and sociodemographic data); 2. telephone interview (specific information on mental health) |
| 18 | Whitaker, M. *et al,* 2022 [47] | England | To estimate  symptom prevalence; to investigate co-occurrence of symptoms and  assess risk factors for persistence of symptoms | Cross-sectional | Mixed population | PCR | Questionnaires and self-reported symptoms |

^a^ All data was extracted by DBR and LOM; ^b^ Due to the number of columns the table was split in two to enable the presentation of all data extracted; PCR: polymerase chain reaction.

**S3 Table: Data extracted from included studies (n=18)^a,b^ *(continuation).***

|  | **Author, year** | **groups of exposure** | **Follow-up points** | **Mean follow-up time (weeks)** | **Day zero of follow-up** | **Mean age** | **Age range** | **Male**  **(n)** | **Mean BMI** | **Evaluated outcomes** | **Date of data extraction** |
| --- | --- | --- | --- | --- | --- | --- | --- | --- | --- | --- | --- |
| 1 | Alkwai, H.M. *et al.,* 2022 [44] | Overweight (BMI >25); Eutrophic (BMI 18.5-24.9). | 1 online survey at least 3 months after covid-19 diagnosis | NA | COVID-19 infection | NA | 18-65 | 51 | NA | Headache, fatigue, loss of smell, body aches, fever, loss of taste, throat pain, muscle pain, bone pain, lower back pain, diarrhoea, loss of appetite, dry cough, runny nose, sleep disturbance, breathing difficulty, mood changes, nausea, muscle weakness, dizziness, chills, inability to concentrate, chest pain, abdominal pain, swallowing difficulty, red itchy eyes and vomiting. | April/2024 |
| 2 | Blümel, J.E. *et al.*, 2022 [53] | Eutrophic (BMI<25);  Overweight (BMI 25-30);  Obesity (BMI>30). | 0 a 18 meses after infection | 32 | Appearance of the infection | 53 | 40-64 | 0 | 25.6 | Fatigability, joint and muscular discomfort, anosmia, shortness of breath, headache, memory impairment, cough, insomnia, taste alteration, hair loss, paraesthesia, anxiety, anorexia, chest pain, vertigo, palpitations, cold and diarrhoea | April/2024 |
| 3 | Bungenberg, J. *et al*.,  2022 [48] | Obesity and non-obesity | 1 visit at least 4 weeks after covid-19 acute diagnosis | 29.3 (range 3.23-57.86) weeks | COVID-19 infection | 50.5 | 22-84 | 22 | NA  (14% of sample with obesity) | Cognitive deficits, fatigue, smell and/or taste disturbance, sleep problems, headache, emotional disturbance, dyspnea, arthalgia, dizziness, hyperhidrosis, hair loss, cough, hearing problems, palpitations, phonophobia, chest pain, visual disturance, vertigo, obstipation, runny nose/nasal congestion, photophobia, orthostatic hypotension, weight changes, sore throat, general symptoms, adbominal pain, voice disturbance, skin rash, temeprature disturbance, nausea, diarrhea, fever | May/2024 |
| 4 | Carter, S.J. *et al.*, 2022 [54] | Normal weight, overweight and obesity | Up to 4 months from a positive test (phone screen and onsite visit) | 12.14 | Positive laboratory test | 55 | 44-66 | 17 female | Cases: 27.9±7.0  Controls:  25.9±4.4 | Gastrointestinal, Hair loss, Sore throat, Fever, Headache, Cognitive impairment, Fatigue, Shortness of breath, Cough, Joint/muscle ache, Loss of taste / smell | May/2024 |
| 5 | Chudzik, M. *et al.*, 2022 [50] | Obesity (BMI>30 kg/m2) Not obese (BMI <30 kg/m2) | The “0” appointment (first visit); follow-up after 3 months. | 28.8 | The “0” appointment (first visit) - at least 14 days after last symptoms of acute covid-19 | 53.8 | 40.3-67.5 | 1,411 | NA | Smell and Taste Disorders | March/2024 |
| 6 | Desgranges, F. *et al*., 2022 [52] | Obesity; Overweight/ obesity and healthy weight | First period: > 3 to 5 months after diagnosis; second period: > 5 to 7 months after diagnosis; and third period: > 7 to 10 months after diagnosis. | 21.43 | Initial visit to ED, screening centre or outpatient clinic | 41 | 31-54 | 157 | 24 | Predefined long-term symptoms (fatigue, muscle weakness, dyspnoea, cough, thoracic pain, smell or taste disorder, blurred vision, headache, memory impairment, loss of balance, numbness, nausea, sleep disorder and hair loss) | March/2024 |
| 7 | Epsi, N.J. *et al.*, 2024 [55] | Normal/underweight (<24.9 kg/m²); overweight (25-29.9 kg/m²); obese (>30 kg/m²). | 1, 2, 3, 6 and 12 months | 24 | First positive test |  | 18-65 | 1,201 | NA  (78% of sample have overweight/obesity) | Sensory cluster (loss of smell and/or taste); Fatigue and difficulty thinking cluster; Difficulty breathing and exercise intolerance cluster. | August/  2024 |
| 8 | Farhanah, N. *et al*., 2022 [42] | Normal weight (BMI 18.5-25); overweight/obese (BMI >25) | Every week during the 1st month; every 2 weeks in the 2st month and 3st month. | NA | Hospital discharge | 48.9 | 18-65 | 55 | 24 | Fatigue, cough, dyspnoea, nausea, sleeplessness, headache, ageusia, joint pain, anosmia, myalgia, cognitive disorder, abdominal pain, tinnitus, chest pain, runny nose, excessive sweating. | June/2024 |
| 9 | Fernández-de- Las-  Peñas, C. *et al, 2021* [51] | Obesity (BMI>30 kg/m2) Not obese (BMI <30 kg/m2) | NA | 28.8 | Hospital discharge | 52 | 37.5-66.5 | 159 | 31 | Post‐COVID symptoms: Fatigue, Dyspnoea on exertion, Dyspnoea rest, Memory loss, Skin rashes, Gastrointestinal disorders – diarrhoea, Cognitive blunting – brain fog, Concentration loss, Ageusia/hypogeusia, Ocular/vision disorders, Tachycardia – palpitations, Musculoskeletal pain, Anosmia/hyposmia, Migraine‐like headache, anxiety/depressive levels and sleep quality | March/2024 |
| *10* | Gaur, R. *et al*., 2022 [41] | Eutrophic (BMI >25); Overweight (BMI 25-30); Obesity (BMI >30). | NA | 15.5 | Unclear; recovery after COVID-19 infection | 48.7 | 18-84 | 62 | 27.18 | Fatigue, dyspnea, musculoskeletal complaints, difficulty sleeping, generalized body ache, abdominal pain, headache, persistent cough, new onset cough, chest pain, vertigo, nausea, epistaxis | April/2024 |
| *11* | Li, Z. *et al*., 2023^d^ [32] | BMI < 18.5; BMI 18.5-22-9; BMI > 23 | 1 year after discharge | 52 | Discharge of the hospital | 50.8 | 36.4-65.2 | 216 | NA  (41.3% of sample with BMI ≥23) | Poor sleep quality; fatigue, Depression And anxiety | March/2024 |
| 12 | Miyazato, Y. *et al.,* 2022 [43] | BMI as continuous quantitative variable | 6 and 12 months | 35.5 | Symptom onset or diagnosis of COVID-19 | 47 | 39-55 | 226 | 23.3 | Persistent symptoms | March/2024 |
| 13 | Moy, F.M. *et al.*, 2022 [40] | Underweight (<18.5kg/m2) & Normal weight (18.5-24.9kg/m2); overweight (25.0–29.9kg/m2) & obese (>30.0kg/m2). | NA | 27.3 | COVID-19 infection | 40.2 | 20-69 | 302 | NA | Mild to severe symptoms of depression (PHQ-9) | March/2024 |
| 14 | Shang, L. *et al.*, 2021^d^ [33] | Excess of weight (>25 kg/m2); eutrophic (≤25 kg/m2) | Interview (presential) | 46.1 | Discharge of the hospital | 53 | 44-61 | 48 | NA | Shortness of breath, fatigue, sleep difficulties, joint pain, smell disorder, diarrhoea, constipation | March/2024 |
| 15 | Sørensen, A.I.V, *et al*., 2022 [45] | normal weight (18.5-24.9kg/m2); overweight (25.0–29.9kg/m2); obese (>30.0kg/m2). | 6, 9 or 12 months after test date | 38 weeks after COVID-19 test | First positive RT-PCR test results | 49 | 49.0 (IQR: 39–60 years) | 25,172 | 25.2 (22.7, 28.5) | N: Headache, smell disorder, taste disorder, vertigo | June/2024 |
| 16 | Van-Wijhe, M. *et al.,* 2022 [46] | normal weight (18.5-24.9kg/m2); overweight (25.0–29.9kg/m2); obese (>30.0kg/m2). | Online survey | 24 | PCR test | 48.2 | 33-63 | 245 | 26.4 | Loss of sense of taste, loss of sense of smell; shortness of breath during rest, talk, and easy and fast walk; tiredness; concentration problems; screen fatigue; long- and short-term memory problems; tightness in the chest; dry cough; headache; muscle pain; dizziness; light and noise sensitivity; nausea; diarrhoea; and tingling feelings in hands and feet | May/2024 |
| 17 | Vassalini, P. *et al.*, 2021 [49] | Obesity; Not obesity. | 1 telephone interview 3 months after the initial hospitalisation | 12 | hospital discharge | 57 | 48-66 | 62 | NA | Depression (PHQ-9 > 9) | June/2024 |
| 18 | Whitaker, M. *et al,* 2022 [47] | Normal weight (18.5-24.9kg/m2); overweight (25.0–29.9kg/m2); obese (>30.0kg/m2). | Round 3-5 (September/ 2020-January/  2021); Round 6 (may/2021) | NA | Self-test | NA | 80% of sample between  25 and 65 years old | 37,600 | NA | N: Headache, memory issues, numbness, smell disorder, taste disorder, vertigo | July/2024 |

^a^ All data was extracted by DBR and LOM; ^b^ Due to the number of columns the table was split in two to enable the presentation of all data extracted; PCR: polymerase chain reaction; NA: Data non-available
