# Supplementary material for: Excess weight is associated with neurological and neuropsychiatric symptoms in post-COVID-19 condition: A systematic review and meta-analysis
Source: PLoS One. 2025 May 7;20(5):e0314892. doi: 10.1371/journal.pone.0314892 (PMC12057935; doi:10.1371/journal.pone.0314892)
Supplement: S4 Table — PTotal: Total population; POB: Population with obesity; PNOB: Non-obesity population; POW: Population with overweight; PEW: population with excess weight; PEUT: Eutrophic population; NSymptomOB: n of individuals with obesity that reported the symptom; NSymptomNOB: n of non-obesity individuals that reported the symptom; NSymptomOW: n of individuals with overweight that reported the symptom; NSymptomEUT: n of eutrophic individuals that reported the symptom; NSymptomEW: n of individuals with excess weight that reported the symptom; NOSymptomOB: n of individuals with obesity that did not report the symptom; NOSymptomNOB: n of non-obesity individuals that did not report the symptom; NOSymptomOW: n of individuals with overweight that did not report the symptom; NOSymptomEUT: n of eutrophic individuals that did not report the symptom; NOSymptomEW: n of individuals with excess weight that did not report the symptom. (DOCX) [file pone.0314892.s004.docx]

**Supporting Information**

Due to the amount of data extracted from included studies to enable the calculation of meta-analysis, authors have organized data according to the evaluated outcomes (persistent neuro-symptoms of Post-Covid-19 Condition/PCC). We have structured one table per symptom, as detailed below:

**S4 Table: Data extracted from included studies for meta-analysis**

1. **Concentration Issues**
2. **Memory Impairment**
3. **Cognitive Issues**
4. **Headache**
5. **Numbness**
6. **Dizziness**
7. **Smell disorder**
8. **Taste disorder**
9. **Smell and taste disorder**
10. **Anxiety**
11. **Depression**
12. **Mood change**
13. **Sleep Disturbance**

**S4 Table: Data extracted from included studies for meta-analysis**

1. **Concentration Issues**

| **Author** | **Ptotal** | **POB** | **PNOB** | **POW** | **PEW** | **PEUT** | **NCon**  **OB** | **NCon**  **NOB** | **NCon**  **OW** | **NCon**  **EUT** | **NCon**  **EW** | **NOCon**  **OB** | **NOCon**  **NOB** | **NOConc**  **OW** | **NOCon**  **EW** | **NOCon**  **EUT** |
| --- | --- | --- | --- | --- | --- | --- | --- | --- | --- | --- | --- | --- | --- | --- | --- | --- |
| Alkwai, H.M. *et al.,* 2022 | 213 |  |  |  | 32 | 181 |  |  |  | 8 | 4 |  |  |  | 28 | 173 |
| Fernández-de- Las-Peñas, C. et al, 2021 | 264 | 88 | 176 |  |  |  | 12 | 16 |  |  |  | 76 | 160 |  |  |  |

PTotal: Total population; POB: Population with obesity; PNOB: Non-obesity population; POW: Population with overweight; PEW: population with excess weight; PEUT: Eutrophic population; NCon OB: *n* of individuals with obesity that reported concentration issues; NCon NOB: *n* of non-obesity individuals that reported concentration issues; Ncon OW: *n* of individuals with overweight that reported concentration issues; Ncon EUT: *n* of eutrophic individuals that reported concentration issues; Ncon EW: *n* of individuals with excess weight that reported concentration issues; NOCon OB: *n* of individuals with obesity that did not report concentration issues; NOCon NOB: *n* of non-obesity individuals that did not report concentration issues; NOcon OW: *n* of individuals with overweight that did not report concentration issues; NOcon EUT: *n* of eutrophic individuals that did not report concentration issues; NOcon EW: *n* of individuals with excess weight that r did not report concentration issues.

**S4 Table: Data extracted from included studies for meta-analysis**

1. **Memory impairment**

| **Author** | **Ptotal** | **POB** | **PNOB** | **POW** | **PEW** | **PEUT** | **NMem**  **OB** | **NMem**  **NOB** | **NMem**  **OW** | **NMem**  **EUT** | **NMem**  **EW** | **NOMem**  **OB** | **NOMem**  **NOB** | **NOMem**  **OW** | **NOMem**  **EW** | **NOMem**  **EUT** |
| --- | --- | --- | --- | --- | --- | --- | --- | --- | --- | --- | --- | --- | --- | --- | --- | --- |
| Alkwai, H.M. *et al.,* 2022 | 213 |  |  |  | 32 | 181 |  |  |  | 7 | 3 |  |  |  | 29 | 174 |
| Blümel, J.E. *et al.*, 2022 | 304 | 41 | 263 | 124 | 165 | 139 | 2 | 10 | 5 | 5 | 7 | 39 | 253 | 119 | 158 | 134 |
| Bungenberg, J. *et al*., 2022 | 50 | 7 | 43 |  |  |  | 6 | 29 |  |  |  | 1 | 24 |  |  |  |
| Fernández-de- Las-Peñas, C. et al, 2021 | 264 | 88 | 176 |  |  |  | 16 | 26 |  |  |  | 72 | 150 |  |  |  |
| Whitaker, M. *et al.*, 2022 | 89325 | 2816 | 8539 | 4028 | 6844 | 4511 | 285 | 821 | 268 | 255 | 553 | 2531 | 8016 | 3760 | 6291 | 4256 |

PTotal: Total population; POB: Population with obesity; PNOB: Non-obesity population; POW: Population with overweight; PEW: population with excess weight; PEUT: Eutrophic population; NMem OB: *n* of individuals with obesity that reported memory impairment; NMem NOB: *n* of non-obesity individuals that reported memory impairment; NMem OW: *n* of individuals with overweight that reported memory impairment; NMem EUT: *n* of eutrophic individuals that reported memory impairment; NMem EW: *n* of individuals with excess weight that reported memory impairment; NOMem OB: *n* of individuals with obesity that did not report memory impairment; NOMemNOB: *n* of non-obesity individuals that did not report memory impairment; NOMem OW: *n* of individuals with overweight that did not report memory impairment; NOMem EUT: *n* of eutrophic individuals that did not report memory impairment; NOMem EW: *n* of individuals with excess weight that did not report memory impairment;

**S4 Table: Data extracted from included studies for meta-analysis**

1. **Cognitive issues**

| **Author** | **Ptotal** | **POB** | **PNOB** | **POW** | **PEW** | **PEUT** | **NCog**  **OB** | **NCog**  **NOB** | **NCog**  **OW** | **NCog**  **EUT** | **NCog**  **EW** | **NOCog**  **OB** | **NOCog**  **NOB** | **NOCog**  **OW** | **NOCog**  **EW** | **NOCog**  **EUT** |
| --- | --- | --- | --- | --- | --- | --- | --- | --- | --- | --- | --- | --- | --- | --- | --- | --- |
| Bungenberg, J. *et al*., 2022 | 50 | 7 | 43 |  |  |  | 6 | 29 |  |  |  | 1 | 14 |  |  |  |
| Carter, S.J. *et al.*, 2022 | 17 | 4 | 13 | 6 | 10 | 7 | 0 | 2 | 1 | 1 | 1 | 4 | 11 | 5 | 9 | 6 |
| Fernández-de- Las-Peñas, C. et al, 2021 | 264 | 88 | 176 |  |  |  | 8 | 12 |  |  |  | 80 | 164 |  |  |  |

PTotal: Total population; POB: Population with obesity; PNOB: Non-obesity population; POW: Population with overweight; PEW: population with excess weight; PEUT: Eutrophic population; NCog OB: *n* of individuals with obesity that reported cognitive issues; NCog NOB: *n* of non-obesity individuals that reported cognitive issues; NCog OW: *n* of individuals with overweight that reported cognitive issues; NCog EUT: *n* of eutrophic individuals that reported cognitive issues; NCog EW: *n* of individuals with excess weight that reported cognitive issues; NOCog OB: *n* of individuals with obesity that did not report cognitive issues; NOCog NOB: *n* of non-obesity individuals that did not report cognitive issues; NOCog OW: *n* of individuals with overweight that did not report cognitive issues; NOCog EUT: *n* of eutrophic individuals that did not report cognitive issues; NOCog EW: *n* of individuals with excess weight that did not report cognitive issues;

**S4 Table: Data extracted from included studies for meta-analysis**

**d) Headache**

| **Author** | **Ptotal** | **POB** | **PNOB** | **POW** | **PEW** | **PEUT** | **NH**  **OB** | **NH**  **NOB** | **NH**  **OW** | **NH**  **EUT** | **NH**  **EW** | **NOH**  **OB** | **NOH**  **NOB** | **NOH**  **OW** | **NOH**  **EW** | **NOH**  **EUT** |
| --- | --- | --- | --- | --- | --- | --- | --- | --- | --- | --- | --- | --- | --- | --- | --- | --- |
| Alkwai, H.M. *et al.,* 2022 | 213 |  |  |  | 32 | 181 |  |  |  | 18 | 3 |  |  |  | 29 | 163 |
| Gaur, R. *et al*., 2022 | 97 | 32 | 1526 | 27 | 59 | 38 | 2 | 2 | 1 | 1 | 3 | 30 | 63 | 26 | 56 | 37 |
| Blümel, J.E. *et al.*, 2022 | 304 | 41 | 263 | 124 | 165 | 139 | 4 | 11 | 8 | 3 | 12 | 37 | 252 | 116 | 153 | 136 |
| Carter, S.J. *et al.*, 2022 | 17 | 4 | 13 | 6 | 10 | 7 | 2 | 5 | 3 | 2 | 5 | 2 | 8 | 3 | 5 | 5 |
| Fernández-de- Las-Peñas, C. et al, 2021 | 264 | 88 | 176 |  |  |  | 7 | 10 |  |  |  | 81 | 166 |  |  |  |
| Farhanah, N. *et al*., 2022 | 104 |  |  |  | 41 | 63 |  |  |  | 2 | 0 |  |  |  | 41 | 61 |
| Sørensen, A.I.V, *et al*., 2022 | 61002 | 9950 | 44549 | 19264 | 29214 | 25285 | 773 | 2531 | 1106 | 1425 | 1879 | 9177 | 42018 | 18158 | 27335 | 23860 |
| Whitaker, M. *et al.*, 2022 | 78566 | 18892 | 58625 | 27986 | 46878 | 30639 | 1239 | 2656 | 1334 | 1322 | 2573 | 17653 | 55969 | 26652 | 44305 | 29317 |

PTotal: Total population; POB: Population with obesity; PNOB: Non-obesity population; POW: Population with overweight; PEW: population with excess weight; PEUT: Eutrophic population; NH OB: *n* of individuals with obesity that reported headache; NH NOB: *n* of non-obesity individuals that reported headache; NH OW: *n* of individuals with overweight that reported headache; NH EUT: *n* of eutrophic individuals that reported headache; NH EW: *n* of individuals with excess weight that reported headache; NH OB: *n* of individuals with obesity that did not report headache; NH NOB: *n* of non-obesity individuals that did not report headache; NOH OW: *n* of individuals with overweight that did not report headache; NOH EUT: *n* of eutrophic individuals that did not report headache; NOH EW: *n* of individuals with excess weight that did not report headache;

**S4 Table: Data extracted from included studies for meta-analysis**

**e) Numbness**

| **Author** | **Ptotal** | **POB** | **PNOB** | **POW** | **PEW** | **PEUT** | **NNum**  **OB** | **NNum**  **NOB** | **NNum**  **OW** | **NNum**  **EUT** | **NNum**  **EW** | **NONum**  **OB** | **NONum**  **NOB** | **NONum**  **OW** | **NONum**  **EW** | **NONum**  **EUT** |
| --- | --- | --- | --- | --- | --- | --- | --- | --- | --- | --- | --- | --- | --- | --- | --- | --- |
| Alkwai, H.M. *et al.,* 2022 | 213 |  |  |  | 32 | 181 |  |  |  | 5 | 1 |  |  |  | 31 | 176 |
| Blümel, J.E. *et al.*, 2022 | 304 | 41 | 263 | 124 | 165 | 139 | 0 | 5 | 2 | 3 | 2 | 41 | 258 | 122 | 163 | 136 |
| Whitaker, M. *et al.*, 2022 | 78566 |  |  |  |  |  | 574 | 1106 | 564 | 542 | 1138 | 18318 | 57519 | 27422 | 45740 | 30097 |

PTotal: Total population; POB: Population with obesity; PNOB: Non-obesity population; POW: Population with overweight; PEW: population with excess weight; PEUT: Eutrophic population; NNum OB: *n* of individuals with obesity that reported numbness; NNum NOB: *n* of non-obesity individuals that reported numbness; NNum OW: *n* of individuals with overweight that reported numbness; NNum EUT: *n* of eutrophic individuals that reported numbness; NNum EW: *n* of individuals with excess weight that reported numbness; NNum OB: *n* of individuals with obesity that did not report numbness; NNum NOB: *n* of non-obesity individuals that did not report numbness; NONum OW: *n* of individuals with overweight that did not report numbness; NONum EUT: *n* of eutrophic individuals that did not report numbness; NONum EW: *n* of individuals with excess weight that did not report numbness.

**S4 Table: Data extracted from included studies for meta-analysis**

**f) Dizziness**

| **Author** | **Ptotal** | **POB** | **PNOB** | **POW** | **PEW** | **PEUT** | **NDiz**  **OB** | **NDiz**  **NOB** | **NDiz**  **OW** | **NDiz**  **EUT** | **NDiz**  **EW** | **NODiz**  **OB** | **NODiz**  **NOB** | **NODiz**  **OW** | **NODiz**  **EW** | **NODiz**  **EUT** |
| --- | --- | --- | --- | --- | --- | --- | --- | --- | --- | --- | --- | --- | --- | --- | --- | --- |
| Alkwai, H.M. *et al.,* 2022 | 213 |  |  |  | 32 | 181 |  |  |  | 3 | 5 |  |  |  | 27 | 178 |
| Gaur, R. *et al*., 2022 | 97 | 32 | 1526 | 27 | 59 | 38 | 0 | 1 | 0 | 1 | 0 | 32 | 64 | 27 | 59 | 37 |
| Blümel, J.E. *et al.*, 2022 | 304 | 41 | 263 | 124 | 165 | 139 | 0 | 2 | 1 | 1 | 1 | 41 | 261 | 123 | 164 | 138 |
| Bungenberg, J. *et al*., 2022 | 50 | 7 | 43 |  |  |  | 0 | 2 |  |  |  | 7 | 41 |  |  |  |
| Sørensen, A.I.V, *et al*., 2022 | 61002 | 9950 | 44549 | 19264 | 29214 | 25285 | 521 | 1650 | 730 | 920 | 1251 | 9429 | 42899 | 18534 | 27963 | 24365 |
| Whitaker, M *et al.*, 2022 | 78566 | 18892 | 58625 | 27986 | 46878 | 30639 | 760 | 1617 | 778 | 839 | 1538 | 18132 | 57008 | 27208 | 45340 | 29800 |

PTotal: Total population; POB: Population with obesity; PNOB: Non-obesity population; POW: Population with overweight; PEW: population with excess weight; PEUT: Eutrophic population; NDiz OB: *n* of individuals with obesity that reported dizziness; NDiz NOB: *n* of non-obesity individuals that reported dizziness; NDiz OW: *n* of individuals with overweight that reported dizziness; NDiz EUT: *n* of eutrophic individuals that reported dizziness; NDiz EW: *n* of individuals with excess weight that reported dizziness; NDiz OB: *n* of individuals with obesity that did not report dizziness; NDiz NOB: *n* of non-obesity individuals that did not report dizziness; NODiz OW: *n* of individuals with overweight that did not report dizziness; NODiz EUT: *n* of eutrophic individuals that did not report dizziness; NODiz EW: *n* of individuals with excess weight that did not report dizziness;

**S4 Table: Data extracted from included studies for meta-analysis**

**g) Smell disorder**

| **Author** | **Ptotal** | **POB** | **PNOB** | **POW** | **PEW** | **PEUT** | **NSD**  **OB** | **NSD**  **NOB** | **NSD**  **OW** | **NSD**  **EUT** | **NSD**  **EW** | **NOSD**  **OB** | **NOSD**  **NOB** | **NOSD**  **OW** | **NOSD**  **EW** | **NOSD**  **EUT** |
| --- | --- | --- | --- | --- | --- | --- | --- | --- | --- | --- | --- | --- | --- | --- | --- | --- |
| Alkwai, H.M. *et al.,* 2022 | 213 |  |  |  | 32 | 181 |  |  |  | 21 | 6 |  |  |  | 26 | 160 |
| Gaur, R. *et al*., 2022 | 97 | 32 | 1526 | 27 | 59 | 38 |  |  |  |  |  |  |  |  |  |  |
| Blümel, J.E. *et al.*, 2022 | 304 | 41 | 263 | 124 | 165 | 139 | 5 | 24 | 8 | 16 | 13 | 36 | 239 | 116 | 152 | 123 |
| Fernández-de- Las-Peñas, C. et al, 2021 | 264 | 88 | 176 |  |  |  | 2 | 12 |  |  |  | 86 | 164 |  |  |  |
| Farhanah, N. *et al*., 2022 | 104 |  |  |  | 41 | 63 |  |  |  | 1 | 0 |  |  |  | 40 | 63 |
| Shang, L. *et al.*, 2021^d^ | 118 | 53 | 65 |  |  |  | 4 | 5 |  |  |  | 49 | 60 |  |  |  |
| Sørensen, A.I.V, *et al*., 2022 | 61002 | 9950 | 44549 | 19264 | 29214 | 25285 | 1131 | 4485 | 2064 | 2781 | 3195 | 8819 | 39704 | 17200 | 26019 | 22504 |
| Whitaker, M *et al.*, 2022 | 78566 | 18892 | 58625 | 27986 | 46878 | 30639 | 1190 | 3121 | 1450 | 1671 | 2640 | 17702 | 55504 | 26536 | 44238 | 28968 |

PTotal: Total population; POB: Population with obesity; PNOB: Non-obesity population; POW: Population with overweight; PEW: population with excess weight; PEUT: Eutrophic population; NSD OB: *n* of individuals with obesity that reported smell disorder; NSD NOB: *n* of non-obesity individuals that reported smell disorder; NSD OW: *n* of individuals with overweight that reported smell disorder; NSD EUT: *n* of eutrophic individuals that reported smell disorder; NSD EW: *n* of individuals with excess weight that reported smell disorder; NSD OB: *n* of individuals with obesity that did not report smell disorder; NSD NOB: *n* of non-obesity individuals that did not report smell disorder; NOSD OW: *n* of individuals with overweight that did not report smell disorder; NOSD EUT: *n* of eutrophic individuals that did not report smell disorder; NOSD EW: *n* of individuals with excess weight that did not report smell disorder;

**S4 Table: Data extracted from included studies for meta-analysis**

**h) Taste Disorder**

| **Author** | **Ptotal** | **POB** | **PNOB** | **POW** | **PEW** | **PEUT** | **NTD**  **OB** | **NTD**  **NOB** | **NTD**  **OW** | **NTD**  **EUT** | **NTD**  **EW** | **NOTD**  **OB** | **NOTD**  **NOB** | **NOTD**  **OW** | **NOTD**  **EW** | **NOTD**  **EUT** |
| --- | --- | --- | --- | --- | --- | --- | --- | --- | --- | --- | --- | --- | --- | --- | --- | --- |
| Alkwai, H.M. *et al.,* 2022 | 213 |  |  |  | 32 | 181 |  |  |  | 14 | 1 |  |  |  | 31 | 167 |
| Blümel, J.E. *et al.*, 2022 | 304 | 41 | 263 | 124 | 165 | 139 | 1 | 4 | 2 | 2 | 3 | 40 | 259 | 122 | 162 | 137 |
| Fernández-de- Las-Peñas, C. et al, 2021 | 264 | 88 | 176 |  |  |  | 7 | 12 |  |  |  | 81 | 164 |  |  |  |
| Farhanah, N. *et al*., 2022 | 104 |  |  |  | 41 | 63 |  |  |  | 2 | 1 |  |  |  | 40 | 61 |
| Sørensen, A.I.V, *et al*., 2022 | 61002 | 9950 | 44549 | 19264 | 29214 | 25285 | 958 | 3818 | 1658 | 2160 | 2616 | 8992 | 40731 | 17606 | 26598 | 23125 |
| Whitaker, M *et al.*, 2022 | 78566 | 18892 | 58625 | 27986 | 46878 | 30639 | 1048 | 2512 | 1245 | 1267 | 2293 | 17844 | 56113 | 26741 | 44585 | 29372 |

PTotal: Total population; POB: Population with obesity; PNOB: Non-obesity population; POW: Population with overweight; PEW: Population with excess weight; PEUT: Eutrophic population; NTD OB: *n* of individuals with obesity that reported taste disorder; NTD NOB: *n* of non-obesity individuals that reported taste disorder; NTD OW: *n* of individuals with overweight that reported taste disorder; NTD EUT: *n* of eutrophic individuals that reported taste disorder; NTD EW: *n* of individuals with excess weight that reported taste disorder; NTD OB: *n* of individuals with obesity that did not report taste disorder; NTD NOB: *n* of non-obesity individuals that did not report taste disorder; NOTD OW: *n* of individuals with overweight that did not report taste disorder; NOTD EUT: *n* of eutrophic individuals that did not report taste disorder; NOTD EW: *n* of individuals with excess weight that did not report taste disorder;

**S4 Table: Data extracted from included studies for meta-analysis**

**i) Smell and Taste Disorder**

| **Author** | **Ptotal** | **POB** | **PNOB** | **POW** | **PEW** | **PEUT** | **NSTD**  **OB** | **NSTD**  **NOB** | **NSTD**  **OW** | **NSTD**  **EUT** | **NSTD**  **EW** | **NOSTD**  **OB** | **NOSTD**  **NOB** | **NOSTD**  **OW** | **NOSTD**  **EW** | **NOSTD**  **EUT** |
| --- | --- | --- | --- | --- | --- | --- | --- | --- | --- | --- | --- | --- | --- | --- | --- | --- |
| Bungenberg, J. *et al*., 2022 | 50 | 7 | 43 |  |  |  | 5 | 19 |  |  |  | 2 | 24 |  |  |  |
| Carter, S.J. *et al.*, 2022 | 17 | 4 | 13 | 6 | 10 | 7 | 1 | 11 | 5 | 6 | 6 | 3 | 2 | 1 | 4 | 1 |
| Chudzik, M. *et al.*, 2022 | 2218 | 692 | 1485 |  |  |  | 22 | 76 |  |  |  | 670 | 1409 |  |  |  |

PTotal: Total population; POB: Population with obesity; PNOB: Non-obesity population; POW: Population with overweight; PEW: Population with excess weight; PEUT: Eutrophic population; NSTD OB: *n* of individuals with obesity that reported smell and taste disorder; NSTD NOB: *n* of non-obesity individuals that reported smell and taste disorder; NSTD OW: *n* of individuals with overweight that reported smell and taste disorder; NSTD EUT: *n* of eutrophic individuals that reported smell and taste disorder; NSTD EW: *n* of individuals with excess weight that reported smell and taste disorder; NOSTD OB: *n* of individuals with obesity that did not report smell and taste disorder; NOSTD NOB: *n* of non-obesity individuals that did not report smell and taste disorder; NOSTD OW: *n* of individuals with overweight that did not report smell and taste disorder; NOSTD EUT: *n* of eutrophic individuals that did not report smell and taste disorder; NOSTD EW: *n* of individuals with excess weight that did not report smell and taste disorder;

**S4 Table: Data extracted from included studies for meta-analysis**

**j) Anxiety**

| **Author** | **Ptotal** | **POB** | **PNOB** | **POW** | **PEW** | **PEUT** | **NAnx**  **OB** | **NAnx**  **NOB** | **NAnx**  **OW** | **NAnx**  **EUT** | **NAnx**  **EW** | **NOAnx**  **OB** | **NOAnx**  **NOB** | **NOAnx**  **OW** | **NOAnx**  **EW** | **NOAnx**  **EUT** |
| --- | --- | --- | --- | --- | --- | --- | --- | --- | --- | --- | --- | --- | --- | --- | --- | --- |
| Blümel, J.E. *et al.*, 2022 | 304 | 41 | 263 | 124 | 165 | 139 | 3 | 2 | 1 | 1 | 4 | 38 | 261 | 123 | 161 | 138 |
| Fernández-de- Las-Peñas, C. et al, 2021 | 264 | 88 | 176 |  |  |  | 14 | 17 |  |  |  | 74 | 159 |  |  |  |
| Li, Z. et al., 2023 | 535 |  |  |  | 221 | 295 |  |  |  | 41 | 36 |  |  |  | 185 | 254 |

PTotal: Total population; POB: Population with obesity; PNOB: Non-obesity population; POW: Population with overweight; PEW: Population with excess weight; PEUT: Eutrophic population; NAnx OB: *n* of individuals with obesity that reported anxiety NAnx NOB: *n* of non-obesity individuals that reported anxiety; NAnx OW: *n* of individuals with overweight that reported anxiety; NAnx EUT: *n* of eutrophic individuals that reported anxiety; NAnx EW: *n* of individuals with excess weight that reported anxiety; NOAnx OB: *n* of individuals with obesity that did not report anxiety; NOAnx NOB: *n* of non-obesity individuals that did not report anxiety; NOAnx OW: *n* of individuals with overweight that did not report anxiety; NOAnx EUT: *n* of eutrophic individuals that did not report anxiety; NOAnx EW: *n* of individuals with excess weight that did not report anxiety.

**S4 Table: Data extracted from included studies for meta-analysis**

**k) Depression**

| **Author** | **Ptotal** | **POB** | **PNOB** | **POW** | **PEW** | **PEUT** | **NDep**  **OB** | **NDep**  **NOB** | **NDep**  **OW** | **NDep**  **EUT** | **NDep**  **EW** | **NODep**  **OB** | **NODep**  **NOB** | **NODep**  **OW** | **NODep**  **EW** | **NODep**  **EUT** |
| --- | --- | --- | --- | --- | --- | --- | --- | --- | --- | --- | --- | --- | --- | --- | --- | --- |
| Fernández-de- Las-Peñas, C. et al, 2021 | 264 | 88 | 176 |  |  |  | 12 | 28 |  |  |  | 76 | 148 |  |  |  |
| Li, Z. et al., 2023 | 535 |  |  |  | 221 | 295 |  |  |  | 48 | 56 |  |  |  | 173 | 239 |
| Moy, F.M. *et al.*, 2022 | 561 |  |  |  | 315 | 246 |  |  |  | 163 | 104 |  |  |  | 152 | 142 |
| Vassalini, P. *et al.*, 2021 | 115 | 5 | 110 |  |  |  | 1 | 17 |  |  |  | 4 | 93 |  |  |  |

PTotal: Total population; POB: Population with obesity; PNOB: Non-obesity population; POW: Population with overweight; PEW: Population with excess weight; PEUT: Eutrophic population; NDep OB: *n* of individuals with obesity that reported depression NDep NOB: *n* of non-obesity individuals that reported depression; NDep OW: *n* of individuals with overweight that reported depression; NDep EUT: *n* of eutrophic individuals that reported depression; NDep EW: *n* of individuals with excess weight that reported depression; NODep OB: *n* of individuals with obesity that did not report depression; NODep NOB: *n* of non-obesity individuals that did not report depression; NODep OW: *n* of individuals with overweight that did not report depression; NODep EUT: *n* of eutrophic individuals that did not report depression; NODep EW: *n* of individuals with excess weight that did not report depression;

**S4 Table: Data extracted from included studies for meta-analysis**

**l) Mood change**

| **Author** | **Ptotal** | **POB** | **PNOB** | **POW** | **PEW** | **PEUT** | **NMood**  **OB** | **NMood**  **NOB** | **NMood**  **OW** | **NMood**  **EUT** | **NMood**  **EW** | **NOMood**  **OB** | **NOMood**  **NOB** | **NOMood**  **OW** | **NOMood**  **EW** | **NOMood**  **EUT** |
| --- | --- | --- | --- | --- | --- | --- | --- | --- | --- | --- | --- | --- | --- | --- | --- | --- |
| Alkwai, H.M. *et al.,* 2022 | 213 |  |  |  | 32 | 181 |  |  |  | 15 | 2 |  |  |  | 30 | 166 |
| Bungenberg, J. *et al*., 2022 | 50 | 7 | 43 |  |  |  | 3 | 5 |  |  |  | 4 | 38 |  |  |  |

PTotal: Total population; POB: Population with obesity; PNOB: Non-obesity population; POW: Population with overweight; PEW: Population with excess weight; PEUT: Eutrophic population; NMood OB: *n* of individuals with obesity that reported mood change NMood NOB: *n* of non-obesity individuals that reported mood change; NMood OW: *n* of individuals with overweight that reported mood change; NMood EUT: *n* of eutrophic individuals that reported mood change; NMood EW: *n* of individuals with excess weight that reported mood change; NOMood OB: *n* of individuals with obesity that did not report mood change; NOMood NOB: *n* of non-obesity individuals that did not report mood change; NOMood OW: *n* of individuals with overweight that did not report mood change; NOMood EUT: *n* of eutrophic individuals that did not report mood change; NOMood EW: *n* of individuals with excess weight that did not report mood change.

**S4 Table: Data extracted from included studies for meta-analysis**

**m) Sleep disturbance**

| **Author** | **Ptotal** | **POB** | **PNOB** | **POW** | **PEW** | **PEUT** | **NSleep**  **OB** | **NSleep**  **NOB** | **NSleep**  **OW** | **NSleep**  **EUT** | **NSleep**  **EW** | **NOSleep**  **OB** | **NOSleep**  **NOB** | **NOSleep**  **OW** | **NOSleep**  **EW** | **NOSleep**  **EUT** |
| --- | --- | --- | --- | --- | --- | --- | --- | --- | --- | --- | --- | --- | --- | --- | --- | --- |
| Alkwai, H.M. *et al.,* 2022 | 213 |  |  |  | 32 | 181 |  |  |  | 14 | 3 |  |  |  | 29 | 167 |
| Gaur, R. *et al*., 2022 | 97 | 32 | 1526 | 27 | 59 | 38 | 4 | 7 | 5 | 2 | 9 | 28 | 58 | 22 | 50 | 36 |
| Blümel, J.E. *et al.*, 2022 | 304 | 41 | 263 | 124 | 165 | 139 | 1 | 6 | 3 | 3 | 4 | 40 | 257 | 121 | 161 | 136 |
| Bungenberg, J. *et al*., 2022 | 50 | 7 | 43 |  |  |  | 2 | 20 |  |  |  | 5 | 23 |  |  |  |
| Fernández-de- Las-Peñas, C. et al, 2021 | 264 | 88 | 176 |  |  |  | 40 | 45 |  |  |  | 48 | 131 |  |  |  |
| Farhanah, N. *et al*., 2022 | 104 |  |  |  | 41 | 63 |  |  |  | 2 | 0 |  |  |  | 41 | 61 |
| Li, Z. *et al*., 2023 | 535 |  |  |  | 221 | 295 |  |  |  | 128 | 113 |  |  |  | 108 | 167 |
| Shang, L. *et al.*, 2021^d^ | 118 |  |  |  | 53 | 65 | 21 | 29 |  |  |  | 32 | 36 |  |  |  |
| Whitaker, M. *et al.*, 2022 | 78566 | 18892 | 58625 | 27986 | 46878 | 30639 | 1804 | 3687 | 1907 | 1780 | 3711 | 17088 | 54938 | 26079 | 43167 | 28859 |

PTotal: Total population; POB: Population with obesity; PNOB: Non-obesity population; POW: Population with overweight; PEW: Population with excess weight; PEUT: Eutrophic population; NSleep OB: *n* of individuals with obesity that reported sleep disturbance; NSleep NOB: *n* of non-obesity individuals that reported sleep disturbance; NSleep OW: *n* of individuals with overweight that reported sleep disturbance; NSleep EUT: *n* of eutrophic individuals that reported sleep disturbance; NSleep EW: *n* of individuals with excess weight that reported sleep disturbance; NOSleep OB: *n* of individuals with obesity that did not report sleep disturbance; NOSleep NOB: *n* of non-obesity individuals that did not report sleep disturbance; NOSleep OW: *n* of individuals with overweight that did not report sleep disturbance; NOSleep EUT: *n* of eutrophic individuals that did not report sleep disturbance; NOSleep EW: *n* of individuals with excess weight that did not report sleep disturbance.
