# Supplementary material for: Excess weight is associated with neurological and neuropsychiatric symptoms in post-COVID-19 condition: A systematic review and meta-analysis
Source: PLoS One. 2025 May 7;20(5):e0314892. doi: 10.1371/journal.pone.0314892 (PMC12057935; doi:10.1371/journal.pone.0314892)
Supplement: S5 Table — (DOCX) [file pone.0314892.s005.docx]

**Supplementary Information**

**S5 Table: Author contact details and requested data^a^.**

|  | **Author** | **E-mail contact** | **Dates of contact** | **Date of response** | **Requested data** |
| --- | --- | --- | --- | --- | --- |
|  | Alkwai, H.M. *et al.,* 2022 [44] | h.alkwai@uoh.edu.sa | 06.03.2024; 16.04.2024 | 16.04.2024 | The frequency (n, %) of persistent symptoms among overweight individuals. |
|  | Blümel, J.E. *et al.*, 2022 [53] | jeblumelm@gmail.com | 07.03.2024 | 10.03.2024 | The frequency (n, %) of symptoms in women with positive PT-PCR COVID-19 according to BMI status. |
|  | Bungenberg, J. *et al*.,  2022 [48] | kreetz@ukaachen.de  jbungenberg@ukaachen.de | 08.05.2024 | 15.05.2024 | The frequency (n, %) of long-term symptoms, among hospitalized and non-hospitalized patients, according to BMI status. |
|  | Carter, S.J. *et al.*, 2022 [54] | stjcarte@iu.edu | 06.05.2024 | 15.05.2024 | The frequency (n, %) of persistent symptoms reported, among SARS-CoV-2 women participants, according to BMI status. |
|  | Farhanah, N. *et al*., 2022 [42] | nurfarhanahams@gmail.com | 15.03.2024;  15.05.2024 | 20.04.2024; 16.05.2024 | - The frequency (n, %) of persistent symptoms of COVID-19 patients after discharge at week 12, according to BMI status.  - Baseline characteristics of your studied sample regarding BMI status. |
|  | Gaur, R. *et al*., 2022 [41] | satyapmr2015@gmail.com | 15.03.2024 | 17.03.2024 | The frequency (n, %) of post-COVID symptoms after three months of COVID-19 infection, according to BMI status. |
|  | Sørensen, A.I.V, *et al*., 2022 [45] | aivs@ssi.dk | 07.05.2024;  21.05.2024 | 08.05.2024;  03.06.2024 | The frequency (n, %) of persistent symptoms among positive COVID-19 patients, according to BMI status, after 6-12 months. |
|  | Vassalini, P. *et al.*, 2021 [49] | paolo.vassalini@uniroma1.it | 08.05.2024 | 13.05.2024 | The frequency (n, %) of depressive symptoms among patients, according to  BMI status. |
|  | Whitaker, M. *et al,* 2022 [47] | matthew.whitaker18@imperial.ac.uk  p.elliott@imperial.ac.uk  Data available at:  https://figshare.com/articles/dataset/  Persistent_symptom_prevalences_  among_participants_with_COVID-19_more_than_12_weeks_  ago_by_BMI_status/26075347 | 09.05.2024;  27.05.2024 | 22.05.2024; 21.06.2024 | The frequency (n, %) of each symptom persisted for 12 weeks or more among participants with history of COVID-19 infection, according to BMI status |

^a^All corresponding authors of included studies were contacted by e-mail by Debora Barbosa Ronca (DBR – [deboraronca@gmail.com](mailto:deboraronca@gmail.com)); BMI: body mass index; PCR: polymerase chain reaction.
