# Supplementary material for: Excess weight is associated with neurological and neuropsychiatric symptoms in post-COVID-19 condition: A systematic review and meta-analysis
Source: PLoS One. 2025 May 7;20(5):e0314892. doi: 10.1371/journal.pone.0314892 (PMC12057935; doi:10.1371/journal.pone.0314892)
Supplement: S6 Table — Excess weight group (BMI ≥ 25 kg/m2); NW: Normal Weight group (BMI < 25 kg/m2); Obesity group (BMI ≥ 30 kg/m2); NOb: Non-obesity group (BMI < 30 kg/m2). (DOCX) [file pone.0314892.s006.docx]

**Supporting Information**

**S6 Table: Frequency of neurological and neuropsychiatric symptoms of Post-COVID-19 Condition from included studies that did not test for statistical differences according to nutritional status**.

| **Author, year** | **Frequency (%) of PCC Symptoms according to**  **nutritional status** | | | |
| --- | --- | --- | --- | --- |
|  | **Excess Weight *versus* Normal Weight** | | | |
|  | **Exposure groups** | | | **Control group** |
| Alway, H.M. *et al.,* 2022^a^ | Concentration issues  Headache  Memory Impairment  Mood changes  Numbness  Sleep disturbance | | 18.2  13.6  13.6  9.1  4.5  13.6 | 9.3  20.9  8.1  17.4  5.8  16.3 |
| Blümel, J.E. *et al.*, 2022^a^ | Anosmia  Anxiety  Headache  Insomnia  Memory impairment  Numbness  Taste alteration  Vertigo | | 7.9  2.4  7.3  2.4  4.2  1.2  1.8  0.6 | 11.5  0.7  2.2  2.2  3.6  2.2  1.4  0.7 |
| Carter, S.J. *et al.*, 2022^a^ | Cognitive deficit  Headache  Loss of taste/smell | | 10  50  60 | 14.2  28  85.7 |
| Farnahan, N. *et al*., 2022^a^ | Ageusia  Anosmia  Headache  Sleep disturbance | | 2.4  0  0  0 | 4  2  4  4 |
| Gaur, R. *et al*., 2022^a^ | Headache  Sleep disturbance  Vertigo | | 5.0  15.2  0 | 1.0  2.0  1.0 |
| Sørensen, A.I.V, *et al*., 2022 ^a^ | Headache  Smell disorder  Taste disorder  Vertigo | | 6.4  10.9  9.0  4.3 | 5.6  11  8.5  3.6 |
| Whitaker, M. *et al,* 2022 | Headache  Memory Impairment  Numbness  Sleep disturbance  Smell disorder  Taste disorder  Vertigo | | 5.5  1.2  2.4  7.9  5.6  4.9  3.3 | 4.3  0.8  1.8  5.8  5.5  4.1  2.7 |
|  | | **Obesity *versus* Non-obesity** | | |
|  | **Exposure groups** | | | **Control group** |
| Blümel, J.E. *et al.*, 2022 ^a^ | Anosmia  Anxiety  Headache  Insomnia  Memory impairment  Numbness  Taste alteration  Vertigo | | 70.7  12.2  36.6  17.1  29.3  12.2  12.2  4.9 | 9.1  0.8  4.2  2.3  3.8  1.9  1.5  0.8 |
| Bungenberg, J. *et al*., 2022 ^a^ | Cognitive deficit  Mood change  Sleep disturbance  Smell and/or taste disorder  Vertigo | | 85  42  46.5  71.4  0 | 67.4  11.6  28.5  44.1  4.6 |
| Carter, S.J. *et al.*, 2022 ^a^ | Cognitive deficit  Headache  Loss of taste/smell | | 0  50  25 | 15.3  38.4  84.6 |
| Gaur, R. *et al*., 2022^a^ | Headache  Sleep disturbance  Vertigo | | 2.0  4.1  0 | 3.0  10.7  1.5 |
| Sørensen, A.I.V, *et al*., 2022 ^a^ | Headache  Smell disorder  Taste disorder  Vertigo | | 7.8  11.4  9.6  5.2 | 5.7  10.9  8.6  3.7 |
| Vassalini, P. *et al.*, 2021 | Depresion | | 20 | 15.5 |
| Whitaker, M. *et al,* 2022 | Headache  Memory Impairment  Numbness  Sleep disturbance  Smell disorder  Taste disorder  Vertigo | | 6.6  1.5  3.0  9.5  6.3  5.5  4.0 | 4.5  0.9  1.9  6.3  5.3  4.3  2.8 |

Excess weight group (BMI ≥ 25kg/m^2^); NW: Normal Weight group (BMI < 25kg/m^2^); Obesity group (BMI ≥ 30kg/m^2^); NOb: Non-obesity group (BMI < 30kg/m^2^).
