# Supplementary material for: Excess weight is associated with neurological and neuropsychiatric symptoms in post-COVID-19 condition: A systematic review and meta-analysis
Source: PLoS One. 2025 May 7;20(5):e0314892. doi: 10.1371/journal.pone.0314892 (PMC12057935; doi:10.1371/journal.pone.0314892)
Supplement: S7 Table — A) Excess weight versus normal weight; B) Obesity versus non-obesity group. CI: confidence interval; OR: odds ratio; RR: risk ratio; a. The risk of bias of included studies is related to the measurement of exposure (self-reported) or the control of confounders, particularly regarding the severity of COVID-19 acute phase; b. There are less than 10 studies included in the metanalyses therefore we were unable to evaluate publication bias;.c. Metanalysis showed substantial heterogeneity among included studies; d. Metanalysis showed moderate heterogeneity among included studies. (DOCX) [file pone.0314892.s007.docx]

**Supporting Information**

**S7 Table. Assessment of the overall certainty of the evidence for the association of neurological and neuropsychiatric symptoms of Post-COVID-19 Condition between exposure and control groups.**

1. **Excess weight *versus* normal weight**

| 1. **Certainty assessment** | | | | | | | **№ of patients** | | **Effect** | | **Certainty** |
| --- | --- | --- | --- | --- | --- | --- | --- | --- | --- | --- | --- |
| **№ of studies** | **Study design** | **Risk of bias** | **Inconsistency** | **Indirectness** | **Imprecision** | **Other considerations** | **Excess weight** | **normal weight** | **Relative (95% CI)** | **Absolute (95% CI)** |  |
| **Smell disorder** | | | | | | | | | | | |
| 5 | non-randomised studies | serious^a^ | not serious | not serious | not serious | publication bias strongly suspected^b^ | 5.854/76.329 (7.7%) | 4.49/56.308 (8.0%) | **RR 1.01** (0.97 to 1.05) | **1 more per 1000** (from 2 fewer to 4 more) | ⨁⨁◯◯ Low^a,b^ |
| **Anxiety** | | | | | | | | | | | |
| 2 | non-randomised studies | serious^a^ | not serious | not serious | not serious | publication bias strongly suspected^b^ | 40/386 (10.4%) | 42/434 (9.7%) | **RR 1.22** (0.81 to 1.82) | **21 more per 1000** (from 18 fewer to 79 more) | ⨁⨁◯◯ Low^a,b^ |
| **Smell and Taste disorder** | | | | | | | | | | | |
| 2 | non-randomised studies | serious^a^ | not serious | not serious | not serious | publication bias strongly suspected^b^ | 0 cases 0 controls / exposed / unexposed | | **OR 0.65** (0.31 to 1.36) | **0 fewer per 1000** (from 0 fewer to 0 fewer) | ⨁⨁◯◯ Low^a,b^ |

**CI:** confidence interval; **OR:** odds ratio; **RR:** risk ratio; a. The risk of bias of included studies is related to measurement of exposure (self-reported) or confounders control, especially regarding severity of COVID-19 acute phase; b. There are less than 10 studies included in the metaanalyses therefore we were unable to evaluate publication bias.

1. **Obesity *versus* non-obesity group**

| **Certainty assessment** | | | | | | | **№ of patients** | | **Effect** | | **Certainty** |
| --- | --- | --- | --- | --- | --- | --- | --- | --- | --- | --- | --- |
| **№ of studies** | **Study design** | **Risk of bias** | **Inconsistency** | **Indirectness** | **Imprecision** | **Other considerations** | **Obesity** | **Non-obesity** | **Relative (95% CI)** | **Absolute (95% CI)** |  |
| **Headache (follow-up: range 12 weeks to 38 weeks; assessed with: Questionnaire)** | | | | | | | | | | | |
| 6 | non-randomised studies | serious^a^ | not serious | not serious | not serious | publication bias strongly suspected^b^ | 7242 cases 134159 controls 2027/29007 exposed 5215/105152 unexposed | | **OR 1.45** (1.37 to 1.53) | **0 fewer per 1000** (from 0 fewer to 0 fewer) | ⨁⨁◯◯ Low^a,b^ |
| **Memory issues (follow-up: range 12 weeks to 32 weeks; assessed with: Questionnaire)** | | | | | | | | | | | |
| 4 | non-randomised studies | serious^a^ | not serious | not serious | not serious | publication bias strongly suspected^b^ | 1195 cases 11973 controls 309/2952 exposed 886/9021 unexposed | | **OR 1.11** (0.97 to 1.28) | **0 fewer per 1000** (from 0 fewer to 0 fewer) | ⨁⨁◯◯ Low^a,b^ |
| **Numbness (follow-up: range 12 weeks to 32 weeks; assessed with: Questionnaires and self-reporte)** | | | | | | | | | | | |
| 2 | non-randomised studies | serious^a^ | not serious | not serious | not serious | publication bias strongly suspected^b^ | 574/18933 (3.0%) | 1111/58888 (1.9%) | **RR 1.61** (1.46 to 1.78) | **12 more per 1000** (from 9 more to 15 more) | ⨁⨁◯◯ Low^a,b^ |
| **Smell disorder (follow-up: range 12 weeks to 46 weeks; assessed with: Questionnaire)** | | | | | | | | | | | |
| 5 | non-randomised studies | serious^a^ | not serious | not serious | not serious | publication bias strongly suspected^b^ | 9979 cases 134260 controls 2332/29056 exposed 7647/105204 unexposed | | **OR 1.16** (1.11 to 1.22) | **0 fewer per 1000** (from 0 fewer to 0 fewer) | ⨁⨁◯◯ Low^a,b^ |
| **Taste disorder (assessed with: Questionnaire and self-reported)** | | | | | | | | | | | |
| 4 | non-randomised studies | serious^a^ | not serious | not serious | serious^c^ | publication bias strongly suspected^b^ | 8360 cases 132584 controls 2014/28971 exposed 6346/103613 unexposed | | **OR 1.22** (1.08 to 1.32) | **0 fewer per 1000** (from 0 fewer to 0 fewer) | ⨁◯◯◯ Very low^a,b,c^ |
| **Vertigo (follow-up: range 12 weeks to 38 weeks; assessed with: Questionnaire and self-reported)** | | | | | | | | | | | |
| 5 | non-randomised studies | serious^a^ | not serious | not serious | not serious | publication bias strongly suspected^b^ | 1281/28922 (4.4%) | 3272/105006 (3.1%) | **RR 1.44** (1.35 to 1.53) | **14 more per 1000** (from 11 more to 17 more) | ⨁⨁◯◯ Low^a,b^ |
| **Anxiety** | | | | | | | | | | | |
| 2 | non-randomised studies | serious^a^ | not serious | not serious | serious^d^ | publication bias strongly suspected^b^ | 36 cases 522 controls 17/112 exposed 19/420 unexposed | | **OR 3.49** (0.65 to 18.71) | **0 fewer per 1000** (from 0 fewer to 0 fewer) | ⨁◯◯◯ Very low^a,b,c^ |
| **Depression** | | | | | | | | | | | |
| 2 | non-randomised studies | not serious | not serious | not serious | not serious | publication bias strongly suspected^b^ | 58 cases 321 controls 13/80 exposed 45/286 unexposed | | **OR 0.87** (0.44 to 1.75) | **0 fewer per 1000** (from 0 fewer to 0 fewer) | ⨁⨁⨁◯ Moderate^b^ |
| **Sleep disturbance** | | | | | | | | | | | |
| 6 | non-randomised studies | serious^a^ | not serious | not serious | serious^e^ | publication bias strongly suspected^e^ | 7438 cases 72684 controls 1872/19133 exposed 5566/59237 unexposed | | **OR 1.41** (0.93 to 2.13) | **0 fewer per 1000** (from 0 fewer to 0 fewer) | ⨁◯◯◯ Very low^a,d^ |
| **Cognitive issues** | | | | | | | | | | | |
| 3 | non-randomised studies | not serious | not serious | not serious | not serious | publication bias strongly suspected^b^ | 48 cases 0 controls 14/99 exposed 43/232 unexposed | | **OR 1.42** (0.62 to 3.27) | **0 fewer per 1000** (from 0 fewer to 0 fewer) | ⨁⨁⨁◯ Moderate^b^ |
|  |  |  |  |  |  |  | - | 0.0% |  |  |  |
| **Smell and taste disorder** | | | | | | | | | | | |
| 4 | non-randomised studies | not serious | not serious | not serious | serious^e^ | publication bias strongly suspected^b^ | 166 cases 4066 controls 37/1413 exposed 129/2819 unexposed | | **OR 0.69** (0.32 to 1.46) | **0 fewer per 1000** (from 0 fewer to 0 fewer) | ⨁⨁◯◯ Low^b,d^ |

**CI:** confidence interval; **OR:** odds ratio; **RR:** risk ratio; a. The risk of bias of included studies is related to the measurement of exposure (self-reported) or the control of confounders, particularly regarding the severity of COVID-19 acute phase; b. There are less than 10 studies included in the metanalyses therefore we were unable to evaluate publication bias;.c. Metanalysis showed substantial heterogeneity among included studies; d. Metanalysis showed moderate heterogeneity among included studies.
