# Supplementary material for: Excess weight is associated with neurological and neuropsychiatric symptoms in post-COVID-19 condition: A systematic review and meta-analysis
Source: PLoS One. 2025 May 7;20(5):e0314892. doi: 10.1371/journal.pone.0314892 (PMC12057935; doi:10.1371/journal.pone.0314892)

**Supporting Information**

**S1 Fig. Forest-plots of the association of excess weight and the risk of neurological and neuropsychiatric symptoms**.

a) Headache
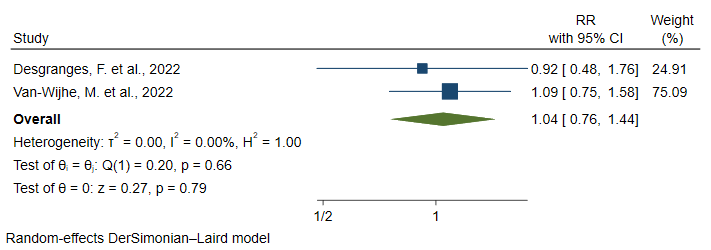


b) Memory issues


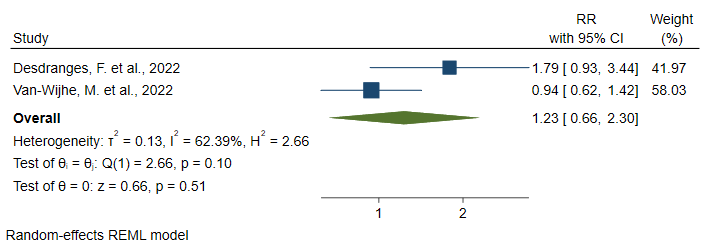


c) Smell disorder


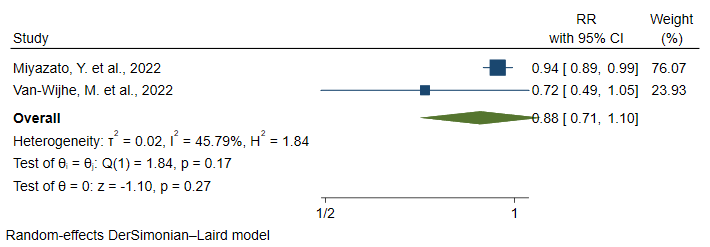


d) Taste disorder


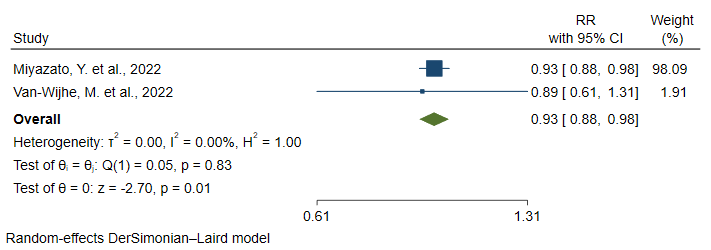

Supplement: S1 Fig — (DOCX) [file pone.0314892.s008.docx]
