# Supplementary material for: Excess weight is associated with neurological and neuropsychiatric symptoms in post-COVID-19 condition: A systematic review and meta-analysis
Source: PLoS One. 2025 May 7;20(5):e0314892. doi: 10.1371/journal.pone.0314892 (PMC12057935; doi:10.1371/journal.pone.0314892)

**Supporting Information**

**S3 Fig. Traffic light plots of risk of bias of included studies that reported the risk of developing neuro-symptoms assessed by Robbins-e tool.**


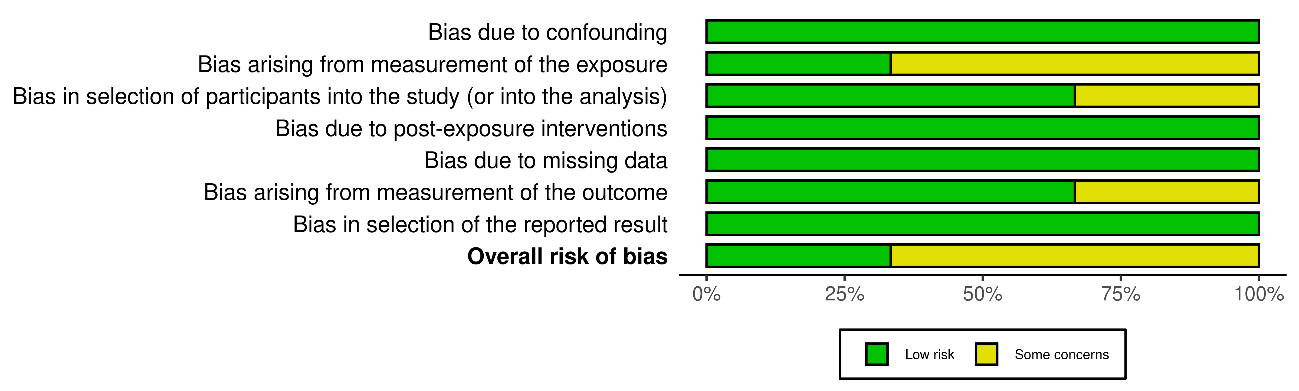


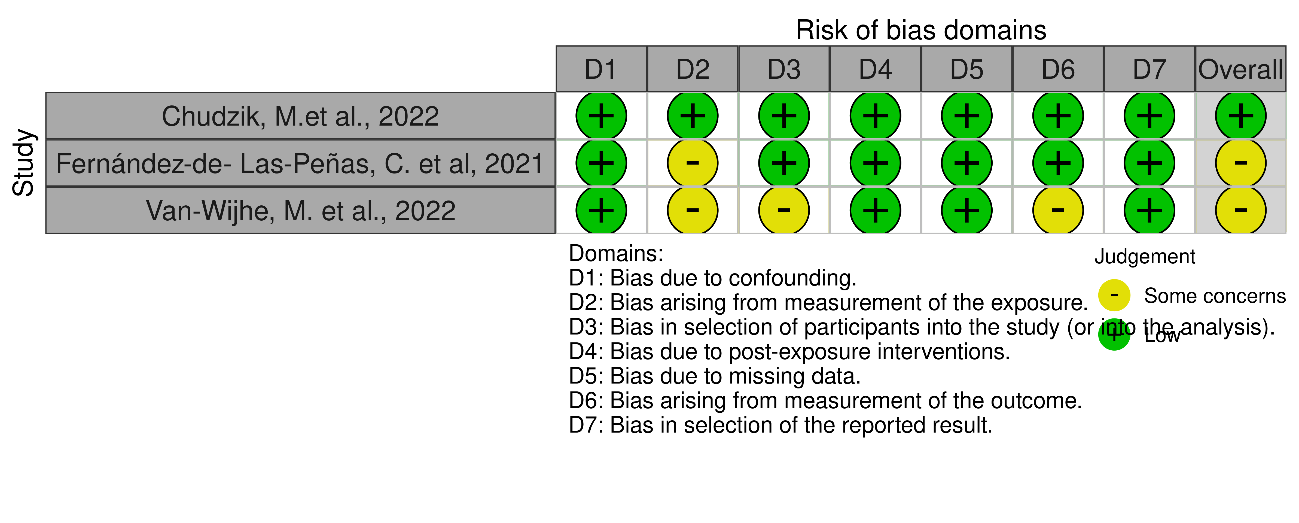

Supplement: S3 Fig — (DOCX) [file pone.0314892.s010.docx]
