# Supplementary material for: Excess weight is associated with neurological and neuropsychiatric symptoms in post-COVID-19 condition: A systematic review and meta-analysis
Source: PLoS One. 2025 May 7;20(5):e0314892. doi: 10.1371/journal.pone.0314892 (PMC12057935; doi:10.1371/journal.pone.0314892)
Supplement: Attachment — Submitted filename: Response to Reviewers.docx [file pone.0314892.s012.docx]

Dong Keon Yon
Academic Editor
PLOS ONE

Nº.: PONE-D-24-52453

Title: Excess weight is associated with persistent neurological and neuropsychiatric symptoms in post-COVID-19 condition: A systematic review and meta-analysis

PLOS ONE

Dear Dr. Dong Keon Yon,

We sincerely appreciate the opportunity to resubmit our article and present a revised version addressing the reviewers’ comments point-by-point. All changes in the original manuscript have been highlighted. We confirm the integrity of the data in this study and hope that we have adequately addressed the requested revisions to your satisfaction.

Best regards,

On behalf of the co-authors,

Débora Ronca

Journal requirements:

If you would like to make changes to your financial disclosure, please include your updated statement in your cover letter. Guidelines for resubmitting your figure files are available below the reviewer comments at the end of this letter.

Response: The financial disclosure has been included in the updated cover letter as follows: “This research received a grant from the Research Support Program at the School of Health Sciences, Brasília, Brazil, funded by the Health Sciences Teaching and Research Foundation (Grant and Acceptance Term No. 5/2020 – FEPECS/DE) (DBR). This study was also partially financed by the Coordenação de Aperfeiçoamento de Pessoal de Nível Superior, Brasil – Finance Code 001 (DBR). MS is supported by the Western Australian Future Health Research and Innovation Fund (Grant ID WANMA/Ideas2023-24/10). KMBC is supported by National Council for Scientific and Technological Development CNPq (Grant n. 302740/2022-8). The funders had no role in the study design, data collection and analysis, decision to publish, or preparation of the manuscript.”

Journal requirements:

When submitting your revision, we need you to address these additional requirements.

1. Please ensure that your manuscript meets PLOS ONE's style requirements, including those for file naming.

Response: The manuscript has been revised to meet PLOS ONE's style requirements, including file naming conventions. We hope this amended version meets the journal's expectations.

2. As required by our policy on Data Availability, please ensure your manuscript or supplementary information includes the following:

- A numbered table of all studies identified in the literature search, including those that were excluded from the analyses.

Response: A new file encompassing all records identified in the literature search, including those excluded from the analyses, has been added to the manuscript as supplementary material (S2 Table). This is also referenced in the main text (see Line 154). We also included this information in our updated cover letter, to highlight our response to the editor’s request after the revised submission. It is worth mentioning that the included file (*.xlxs* format) has two sheets, named “Databases” and “Citation searching”. The “Databases” sheet encompasses 10,122 records identified from databases and the “Citation searching” sheet encompasses 30 studies identified from citation searching. For all the excluded studies, the reasons for exclusion were described. Moreover, an updated PRISMA flowchart was uploaded as figure 1 due to a minor typing error identified (instead of 10,125 records screened from databases, 10,122 records were screened – as indicated in the abstract (line 38) and results sessions (line 234).

- For every excluded study, the table should list the reason(s) for exclusion.

Response: A file listing all studies identified in the literature search, along with the reasons for exclusion, has been added to the manuscript (S2 Table and Line 154).

- If any of the included studies are unpublished, include a link (URL) to the primary source or detailed information about how the content can be accessed.

Response: All included studies have been previously published or are available as preprints.

A table of all data extracted from the primary research sources for the systematic review and/or meta-analysis. The table must include the following information for each study:

- Name of data extractors and date of data extraction;
- Confirmation that the study was eligible to be included in the review;
- All data extracted from each study for the reported systematic review and/or meta-analysis that would be needed to replicate your analyses.

Response: Supplementary files (S3 Table and S4 Table) containing the data extracted from the included studies, along with the names of data extractors, extraction dates, eligibility confirmation, and all relevant data, have been included with the manuscript (S3 Table, S4 Table, and Line 188).

If data or supporting information were obtained from another source (e.g. correspondence with the author of the original research article), please provide the source of data and dates on which the data/information were obtained by your research group.

Response: A supplementary file (S5 Table) with details on the type and dates of data obtained from another source has been included in the manuscript (S5 Table and Line 189).

If applicable for your analysis, a table showing the completed risk of bias and quality/certainty assessments for each study or outcome.  Please ensure this is provided for each domain or parameter assessed. For example, if you used the Cochrane risk-of-bias tool for randomized trials, provide answers to each of the signalling questions for each study. If you used GRADE to assess certainty of evidence, provide judgements about each of the quality of evidence factor. This should be provided for each outcome.

Response: The reviewer is right about the importance of presenting the risk of bias and GRADE for each study, including all items of the instruments and for each outcome. We applied the ROBBINS-E tool to assess the risk of bias for the included studies, taking into account both frequency and odds ratio measures of effect. The completed risk of bias assessment is shown in Fig. 6 and S3 Fig. Additionally, we used the GRADE tool to assess the quality/certainty of evidence for all investigated outcomes (symptoms), as presented in Table 3 and S7 Table. It is worth mentioning that Table 3 presents the certainty of evidence for the main outcomes evaluated by GRADE, according to Cochrane recommendations, which emphasize patient-important outcomes (Cochrane Handbook 2012, Section 5.4a).

An explanation of how missing data were handled.

Response: This systematic review included studies that reported the frequency (n, %) of persistent neurological and neuropsychiatric symptoms associated with post-Covid-19 condition (PCC), categorized by nutritional status among COVID-19 survivors. Studies were eligible if they reported data on one or more symptoms of the evaluated outcomes. The meta-analyses were conducted using all available data for each symptom, with separate analyses for each outcome. Corresponding authors were contacted to share data on symptom frequencies by nutritional status (S6 Table). For studies that did not report data for a specific symptom, that study was excluded from the corresponding meta-analysis, ensuring that missing data was not an issue. As such, no imputation of missing data was necessary. Additionally, due to the limited number of studies reporting the same outcome (e.g., anxiety, headache) across exposure/control groups (obesity vs. non-obesity, excess weight vs. normal weight), we were unable to perform the Egger’s test to evaluate publication bias. (Lines 229-230). Finally, considering the differences in symptoms terminology among eligible studies and issues regarding how missing data were handled we included as limitation of our study that this inconsistency in naming PCC symptoms might have led to underestimation of results due to limited data of persistent symptoms according to nutritional status (Lines 513-515).

This information can be included in the main text, supplementary information, or relevant data repository. Please note that providing these underlying data is a requirement for publication in this journal, and if these data are not provided your manuscript might be rejected.

Response: We ensure that we have provided all the underlying data required for publication in this journal.

3. Please include a caption for figure 3.

Answer: An appropriate caption has been included for Fig 3 (Lines 308-309). The figure 3 is also referred in the text (line 300).

 Additional Editor Comments:

Please address the excellent comments from the reviewers.
Response: Indeed, the reviewers' comments were excellent, which contributed to the improvement of the manuscript. We are grateful for that.

[Note: HTML markup is below. Please do not edit.]

Reviewers' comments:
Reviewer's Responses to Questions

**Comments to the Author**

5. Review Comments to the Author
Please use the space provided to explain your answers to the questions above. You may also include additional comments for the author, including concerns about dual publication, research ethics, or publication ethics. (Please upload your review as an attachment if it exceeds 20,000 characters)

Reviewer #1: This systematic review and meta-analysis studied the association between excess weight and post-acute neuropsychiatric sequelae of COVID-19. Despite conducting a rigorous research study, drawing the cause-and-effect relationship between excess weight and post-acute neuropsychiatric sequelae of COVID-19 is statistically inappropriate due to the inclusion of cross-sectional studies. I also suggest using odds ratios instead of relative risks. Other comments are as follows:

Answer: We appreciate the reviewer’s comments. As suggested, odds ratios (ORs) are commonly reported in cross-sectional studies when logistic regression is used to assess the association between risk factors and outcome while controlling for confounders. When the outcome is rare (<10%), the OR and risk ratio (RR) are approximately equal, and the OR can be used to approximate the RR (allowing the OR to serve as an approximation of the RR) (Ref.: Wilber ST & Fu R., 2010). We acknowledge as a limitation of our study that the inclusion of cross-sectional studies limits the ability to establish causal relationships (Lines 509-510). However, our data reveals a significant association between excess weight and persistent neurological and neuropsychiatric symptoms in post-COVID-19 Condition. In response to the reviewer’s suggestion, we have revised the manuscript to replace terms related to “*risk*” with “*is associated with/association*” for greater clarity. The title of the manuscript was also updated to avoid misunderstandings. Additionally, to address the concern about statistical appropriateness, we reviewed all pooled analysis involving case-control included studies (Carter SJ, *et al.*, 2022 and Fernandes-De-Las-Penas C, et al. 2021) and recalculated the results as *Odds Ratios (ORs)* instead of *Risk Ratios (RRs)*. Specifically, pooled ORs were calculated for the following comparisons: obesity *versus* non-obesity groups for cognitive impairment, headache, memory issues, smell disorder, taste disorder, smell and taste disorder, anxiety, depression and sleep disturbance; and excess weight *versus* normal weight for headache, smell and taste disorder. We have also updated the Methods section to clarify our approach: “We performed meta-analytic calculations using STATA software (SE/17). Pooled risk ratios (RRs) with their 95% confidence intervals (CIs) were computed from the raw data of included cohort and cross-sectional studies, while pooled odds ratios (OR) were computed for reported symptoms identified in case-control studies” (Lines 217-220).

References:

1. Wilber ST, Fu R. Risk ratios and odds ratios for common events in cross-sectional and cohort studies. Acad Emerg Med. 2010 Jun;17(6):649-51. doi: 10.1111/j.1553-2712.2010.00773.x. PMID: 20624147.
2. Fernández‐de‐las‐Peñas C, Torres‐Macho J, Elvira‐Martínez CM, Molina‐Trigueros LJ, Sebastián‐Viana T, Hernández‐Barrera V. Obesity is associated with a greater number of long‐term post‐COVID symptoms and poor sleep quality: A multicentre case‐control study. *Int J Clin Pract* 2021; 75: e14917.
3. Carter SJ, Baranauskas MN, Raglin JS, Pescosolido BA, Perry BL. Functional Status, Mood State, and Physical Activity Among Women With Post-Acute COVID-19 Syndrome. *Int J Public Health* 2022; 67: 1604589.

- Page 3 line 61: "Although PCC has no uniform definition in the literature". PCC has a universal definition according to the National Academies of Sciences, Engineering, and Medicine (N Engl J Med. 2024 Nov 7;391(18):1746-1753.)

Response: We appreciate the reviewer’s comment. While a universal definition of PCC was proposed by the National Academies of Sciences, Engineering, and Medicine in November 2024, various broad and non-specific definitions were used in the literature to capture the diverse manifestations of PCC. For instance, the Centers for Disease Control and Prevention (CDC) defined as any signs, symptoms, or conditions persisting for at least four weeks after infection, whereas the World Health Organization (WHO) defined it as the continuation or development of new, otherwise unexplained symptoms three months after the initial SARS-CoV-2 infection. All included studies followed different criteria and definitions of PCC, as the universal definition by the National Academies of Sciences, Engineering, and Medicine was published only in November 2024. Thus, we updated our manuscript and included this important reference in our introduction sections. Moreover, considering the available references during the time of our study and the proposed universal definition, we changed the phrase “Although PCC has no uniform definition in the literature” to “Although PCC has varying definition in the literature” (line 61) and also included information regarding varying terminologies in lines 103-107.

References:

WHO. A clinical case definition of post-COVID-19 condition by a Delphi consensus - The Lancet Infectious Diseases, https://www.thelancet.com/journals/laninf/article/PIIS1473-3099(21)00703-9/fulltext (accessed 15 October 2023).

- Page 9 line 214: "Statistical heterogeneity was assessed using the I2 statistic". How was substantial heterogeneity defined? This will affect your decision on downgrading the certainty of evidence due to the inconsistency of the results.

Response: We defined substantial heterogeneity based on the thresholds recommended by Cochrane: 0%-40% (no important heterogeneity), 30%-60% (moderate heterogeneity), 50%-90% (substantial heterogeneity), and 75-100% (considerable heterogeneity). This information was included in the Methods section of the revised manuscript (Line 225-228). To determine the degree of heterogeneity, we considered the *I*^2^ value, visual inspection of forest plot, the confidence interval, the direction of the effects, and the clinical and methodological differences among the included studies.

References:

Higgins J, Thomas J, Chandler J, Cumpston M, Li T, Page M, et al. Cochrane Handbook for Systematic Reviews of Interventions, https://training.cochrane.org/handbook/current (accessed 12 June 2024).

Reviewer #2: Major Comments

1. Provide a detailed discussion on the clinical applicability of the findings to post-COVID-19 care for patients with excess weight.

Response: We have expanded the Discussion section of the manuscript to include a detailed analysis of the clinical applicability of our findings to post-COVID-19 care for patients with excess weight (See below or Lines 549-557).

“This systematic review and meta-analysis contribute to the growing body of literature on post-COVID-19 condition by detailing the range of neurological and neuropsychiatric symptoms experienced by adults with both post-COVID-19 condition and excess weight. By revealing these associations, our study underscores the significant physical and mental health burden faced by COVID-19 survivors with excess weight and obesity, offering critical insight to guide rehabilitation efforts. Therefore, the management of EW should be considered in the treatment of neurological and neuropsychiatric symptoms due to the significant impact that these combined conditions have on individuals’ health.”

2. Justify the exclusion of studies focusing on comorbidities and their potential influence on the outcomes.

Response: The PECO framework was defined as follows: **P**opulation: Adults with post-COVID-19 Condition (PCC); **E**xposure: Individuals with excess weight; **C**ontrol: Individuals without excess weight; **O**utcome: Neurological and neuropsychiatric symptoms of PCC. Our focus was on the general population of COVID-19 survivors. Studies that examined long-term COVID-19 sequelae in populations with specific comorbidities (e.g., metastatic cancer patients) were excluded. These studies were not considered eligible because they involved vulnerable groups where the effects of excess weight on outcomes could be confounded by the comorbidities themselves, rather than solely reflecting the impact of obesity. Including such studies could introduce bias by altering the direction or magnitude of the association between excess weight and persistent neuropsychiatric symptoms. By excluding these studies, we ensured that our analysis remained focused on the general population of COVID-19 survivors, allowing for an accurate assessment of the association between excess weight and persistent neuro-symptoms. We updated the method section with this information (Lines 143-146).

3. Elaborate on the impact of high risk of bias and heterogeneity, as highlighted in the ROBINS-E and GRADE assessments.

Response: We have included this information in the Discussion section of the manuscript. Specifically, we addressed how the high risk of bias, as assessed by the ROBINS-E tool, and the low and very low certainty of evidence observed in some analyses highlight the need for caution when drawing conclusions and applying the results to clinical practice. (Lines 496-506).

4. Perform subgroup analyses for demographic variables (e.g., age, sex) and COVID-19 severity to refine interpretations.

Response: Subgroup analyses could not be performed due to the limited number of studies included in each meta-analysis. Most of the meta-analyses contained only 4 to 6 studies, with the higher number of studies included for sleep disturbance and headache (9 studies each). Subgroup analysis is typically recommended when at least 10 studies are available to ensure statistical power and meaningful interpretation. Consequently, the available data did not allow for reliable subgroup analyses based on demographic variables (e.g., age, sex, follow-up period) or COVID-19 severity. This limitation is acknowledged in the Discussion (Lines 522-523).

5. Address methodological limitations of included studies, particularly regarding exposure measurement and confounder control.

Response: The methodological limitations of the included studies, particularly concerning exposure measurement and confounder control, have been addressed in the Discussion section. We highlighted the variability in defining and measuring excess weight (Lines 477-480), the lack of standardized approaches to assessing long COVID symptoms (Lines 492-494), the limited adjustment for potential confounders in many studies (Lines 488-492), and study design (Lines 509-510). The Discussion has been updated to reflect these issues more comprehensively, emphasizing their potential impact on the validity and generalizability of the findings.

6. Clarify the choice of random-effects models and provide details on the handling of missing data in the meta-analyses.

Response: We adopted a random-effects model due to the methodological heterogeneity among included studies, particularly concerning the study design, and exposure and outcome measurements. This model is conservative and accounts for between-study variability, producing wider confidence intervals that provide a more cautious estimation of the effect size. By incorporating heterogeneity among studies, the random-effects model gives a more generalized and cautious summary estimate compared to the fixed-effect model. To maximize the data included in our meta-analysis, we contacted the corresponding authors of studies meeting eligibility criteria to request additional information on the frequency of neuro-symptoms by nutritional status among COVID-19 survivors if such data were not presented in the original publication. Data imputation was not performed, as we did not attempt to handle missing data. Instead, we included all available data for each outcome in the analysis. Studies that did not report sufficient data were excluded from the meta-analysis for the respective outcomes. This approach ensured the integrity of the findings while maintaining transparency in our data-handling process.

7. Integrate findings with the broader literature on post-COVID-19 conditions and obesity.
Response: We have updated the Discussion section to include recent studies on post-COVID-19 conditions (PCC) and obesity (Lines 416-419; 427-437; 437-441; 456-459; 535-538). These studies help contextualize our findings within the broader literature by highlighting how obesity may exacerbate or contribute to persistent neuropsychiatric symptoms in COVID-19 survivors. By synthesizing our results with the current body of knowledge, we provide a more comprehensive understanding of the interplay between excess weight and post-COVID-19 outcomes, helping to inform clinical practices and future research directions.

Minor Comments

1. Ensure figure captions are precise and consistent with the content.

Response: All figures and captions have been reviewed to ensure they are precise and consistent with the content presented in the manuscript.

2. Clearly define terms such as "suboptimal health condition" with appropriate citations.
Response: Health, as defined by the World Health Organization, is “a state of complete physical, mental, and social well-being and not merely the absence of disease or infirmity”. However, a growing number of individuals worldwide report experiencing general malaise even in the absence of a diagnosable disorder. These individuals may complain of poor healthy perception without meeting the diagnostic criteria for specific diseases. This intermediate state between optimal health and diagnosable diseases is referred to as suboptimal health status (SHS) (Wang, W., Yan, Y., Guo, Z. *et al.,* 2021). Both underweight and overweight are classified as suboptimal body weight. Excess weight, encompassing both overweight and obesity, is a major risk factor for various chronic conditions. Overweight, defined as excess body weight relative to a healthy standard, poses a risk to health, while obesity is considered a metabolic disease. We have updated the reference to reflect this definition accordingly.

Reference:

Wang, W., Yan, Y., Guo, Z. *et al.* All around suboptimal health — a joint position paper of the Suboptimal Health Study Consortium and European Association for Predictive, Preventive and Personalised Medicine. *EPMA Journal* **12**, 403–433 (2021). https://doi-org.ezproxy.ecu.edu.au/10.1007/s13167-021-00253-2)

3. Streamline data presentation in tables and shift less critical details to supplementary materials.
Response: We have included less critical data as supplementary information as suggested by the reviewer.

4. Expand on ethical considerations, particularly for studies relying on self-reported data.

Response: All included studies adhered to ethical guidelines and were approved by their respective ethical committees. We included this information in the Methods section (Lines 147-148). However, it is important to note the potential limitations associated with studies relying on self-reported data. Self-reported data are subject to recall bias, social desirability bias, and variations in individual interpretation of symptoms, which may affect the accuracy of the findings. Future research should aim to complement self-reported data with objective clinical assessments to enhance the validity of the results and address these potential biases. We have incorporated this information into the Discussion section (Lines 480-482).

5. Update references to include recent, high-impact studies relevant to the research question.

Response: We have updated the Introduction, Discussion, and References sections to include recent, high-impact studies relevant to the research question, as suggested by the reviewer. The added references are detailed below:

Added references:

1. Ely, E. Wesley, Lisa M. Brown, and Harvey V. Fineberg. "Long covid defined." *New England Journal of Medicine* 391.18 (2024): 1746-1753
2. Zeraatkar, D., Ling, M., Kirsh, S., Jassal, T., Shahab, M., Movahed, H., ... & Busse, J. W. (2024). Interventions for the management of long covid (post-covid condition): living systematic review. *bmj*, *387*.
3. Jassat W, Reyes LF, Munblit D, et al, Long COVID in low-income and middle-income countries: the hidden public health crisis. *Lancet* 2023;402:1115-7. doi:10.1016/S0140-6736(23)01685-9
4. Jang S, Hong W, Moon Y. Obesity-compromised immunity in post-COVID-19 condition: a critical control point of chronicity. Front Immunol. 2024 Aug 12;15:1433531. doi: 10.3389/fimmu.2024.1433531. PMID: 39188722; PMCID: PMC11345197.
5. Saccon TD, Mousovich-Neto F, Ludwig RG, Carregari VC, Dos Anjos Souza AB, Dos Passos ASC, Martini MC, Barbosa PP, de Souza GF, Muraro SP, Forato J, Amorim MR, Marques RE, Veras FP, Barreto E, Gonçalves TT, Paiva IM, Fazolini NPB, Onodera CMK, Martins Junior RB, de Araújo PHC, Batah SS, Viana RMM, de Melo DM, Fabro AT, Arruda E, Queiroz Cunha F, Cunha TM, Pretti MAM, Smith BJ, Marques-Souza H, Knittel TL, Ruiz GP, Profeta GS, Fontes-Cal TCM, Boroni M, Vinolo MAR, Farias AS, Moraes-Vieira PMM, Bizzacchi JMA, Teesalu T, Chaim FDM, Cazzo E, Chaim EA, Proença-Módena JL, Martins-de-Souza D, Osako MK, Leiria LO, Mori MA. SARS-CoV-2 infects adipose tissue in a fat depot- and viral lineage-dependent manner. Nat Commun. 2022 Sep 29;13(1):5722. doi: 10.1038/s41467-022-33218-8. PMID: 36175400; PMCID: PMC9521555.
6. Fernández-Pedruelo D, Juárez-Vela R, Ruiz de Viñaspre-Hernández R, Alonso-Alonso J, Criado-Gutiérrez JM and Sancho-Sánchez C (2024) Influence of smoking and obesity on post-COVID-19 sequelae and risk of hospitalization. *Front. Med.* 11:1499239. doi: 10.3389/fmed.2024.1499239

6. Ensure the funding statement explicitly addresses the minimization of funder influence.

Response: The Funding statement has been included in the updated cover letter, and we highlight that funders had no role in study design, data collection and analysis, decision to publish, or preparation of the manuscript”.

Figures and Data

1. Standardize formatting across all forest plots to enhance clarity and interpretability.

Response: A standardized format was applied across all forest plots to improve clarity and interpretability. The updated version includes consistent axis labeling, font sizes, and layout for better readability and uniformity. (Fig. 3; Figs. 4A, 4B, and 4C; Figs. 5A, 5B, and 5C)

2. Include additional plots (e.g., funnel plots, sensitivity analyses) to assess and mitigate publication bias.

Response: Publication bias was not assessed due to the limited number of studies included in each meta-analysis. We updated this information in Methods section (Line 229-230) and addressed in the Discussion section. Specifically, Edger’s test was deemed inappropriate for evaluating the publication bias, as the small number of studies could distort the results. The inability to assess publication bias also reduced the certainty of evidence across all evaluated outcomes and highlighted the significant knowledge gap regarding risk factors associated with PCC. (Lines 498-503.
