# Supplementary material for: Excess weight is associated with neurological and neuropsychiatric symptoms in post-COVID-19 condition: A systematic review and meta-analysis
Source: PLoS One. 2025 May 7;20(5):e0314892. doi: 10.1371/journal.pone.0314892 (PMC12057935; doi:10.1371/journal.pone.0314892)
Supplement: Attachment — Submitted filename: Response_to_Reviewers_auresp_2.docx [file pone.0314892.s013.docx]

Dong Keon Yon, MD, FACAAI, FAAAAI
Academic Editor
PLOS ONE

PONE-D-24-52453R1

Title: Excess weight is associated with persistent neurological and neuropsychiatric symptoms in post-COVID-19 condition: A systematic review and meta-analysis

PLOS ONE

Dear Dr. Dong Keon Yon,

We sincerely appreciate the opportunity to resubmit our article and present a revised version addressing the editor’s comments point-by-point. All changes have been highlighted. We confirm the integrity of the data in this study and hope that we have adequately addressed the requested revisions to your satisfaction.

Best regards,

On behalf of the co-authors,

Débora Ronca

Journal requirements:

Please review your reference list to ensure that it is complete and correct. If you have cited papers that have been retracted, please include the rationale for doing so in the manuscript text, or remove these references and replace them with relevant current references. Any changes to the reference list should be mentioned in the rebuttal letter that accompanies your revised manuscript. If you need to cite a retracted article, indicate the article’s retracted status in the References list and also include a citation and full reference for the retraction notice.

Response: We carefully reviewed our reference list and made the necessary corrections. We identified five studies with published errata and/or corrections. In all cases, the authors confirmed that these were minor errors that did not affect the studies’ findings or conclusions. These studies were published in prestigious scientific journals (*The New England Journal of Medicine*, *Nature Reviews Microbiology*, *The Lancet* and *PLOS ONE, General Hospital Psychiatry*). Accordingly, we updated our reference list to reflect this information, as detailed below. Both the original and updated reference lists have been included in the cover letter, with the corrected references highlighted. We confirm that we did not cite any retracted papers.

References updated:

1) Blumenthal D *et al.*, 2020.

**Original:** Blumenthal D, Fowler EJ, Abrams M, Collins SR. Covid-19 — Implications for the Health Care System. *N Engl J Med* 2020; 383: 1483–1488.

**Updated:** Blumenthal D, Fowler EJ, Abrams M, Collins SR. Covid-19 — Implications for the Health Care System. [published correction appears in N Engl J Med. 2020 Oct 22;383(17):1698. *N Engl J Med* 2020; 383: 1483–1488.

2) Davis HE *et al.*, 2023

**Original:** Davis HE, McCorkell L, Vogel JM, Topol EJ. Long COVID: major findings, mechanisms and recommendations. *Nat Rev Microbiol* 2023; 21: 133–146.

**Correction reference included:** Davis HE, McCorkell L, Vogel JM, Topol EJ. Author Correction: Long COVID: major findings, mechanisms and recommendations. *Nat Rev Microbiol* 2023; 21: 408–408.

3) Swinburn BA, *et al., 2019*

**Original:** Swinburn BA, Kraak VI, Allender S, Atkins VJ, Baker PI, Bogard JR, et al. The Global Syndemic of Obesity, Undernutrition, and Climate Change: The Lancet Commission report. *Lancet Lond Engl*. 2019 393(10173):791–846. DOI: 10.1016/S0140-6736(18)32822-8.

**Errata reference included**: Errata in: Department of Error. *Lancet Lond Engl*. 2019 393(10173):746. DOI: 10.1016/S0140-6736(19)30384-8.

4) Hassan NM, *et al.,* 2023

**Original:** Hassan NM, Salim HS, Amaran S, Yunus NI, Yusof NA, Daud N, et al. Prevalence of mental health problems among children with long COVID: A systematic review and meta-analysis. *PLOS ONE*. 2023 18(5):e0282538. DOI: 10.1371/journal.pone.0282538.

**Correction reference included**: PLOS ONE Staff. Correction: Prevalence of mental health problems among children with long COVID: A systematic review and meta-analysis. PLoS One. 2023 Dec 14;18(12):e0296160. doi: 10.1371/journal.pone.0296160. Erratum for: PLoS One. 2023 May 17;18(5):e0282538. doi: 10.1371/journal.pone.0282538. PMID: 38096220; PMCID: PMC10721052.

5) van der Feltz-Cornelis C, *et al.,* 2024.

**Original**: van der Feltz-Cornelis C, Turk F, Sweetman J, Khunti K, Gabbay M, Shepherd J, et al. Prevalence of mental health conditions and brain fog in people with long COVID: A systematic review and meta-analysis. *Gen Hosp Psychiatry*. 2024 88:10–22. DOI: 10.1016/j.genhosppsych.2024.02.009.

**Correction reference included**: van der Feltz-Cornelis CM, Turk F, Sweetman J, Khunti K, Gabbay M, Shepherd J, et al. Corrigendum to ‘Prevalence of mental health conditions and brain fog in people with long COVID: A systematic review and meta-analysis’ [General Hospital Psychiatry volume 88 (2024)10-22 10.1016/j.genhosppsych.2024.02.009]. *Gen Hosp Psychiatry*. 2025 92:112. DOI: 10.1016/j.genhosppsych.2024.09.006.

Additional Editor Comments:

Please see my minor comments

#1. Studies published up to July 2023 were searched independently across eight electronic databases (PubMed ..... Please describe it) to evaluate the risk of developing...

Response: This sentence was included in the original version of the manuscript but was updated in the revised version. It was replaced with the following: *“We conducted a comprehensive search of eight databases, including PubMed and Embase, for studies published up to July 2023.”* To meet the editor’s requirements, we explicitly listed the eight databases in the abstract (lines 32–34): *“We conducted a comprehensive search of eight databases (PubMed, Embase, SCOPUS, Web of Science, VHL, Google Scholar, ProQuest, and medRxiv) for studies published up to July 2023.”* The discrepancy may have occurred because both the original and revised manuscripts were included in the revised submission, and we did not withdraw the original version. We apologize for any confusion and appreciate the opportunity to clarify. We hope that the updated sentence aligns with the editor’s comments.

#2. post-COVID infection -> post-COVID-19 condition or SARS-CoV-2 infection

Response: We change the sentence accordingly (line 73).

#3. I am simply curious—what is the reason for the omission of this particular paper?
<https://pubmed.ncbi.nlm.nih.gov/38918517/>

Response: Although this well-designed study aimed to explore the risk of neuropsychiatric complications following a COVID-19 diagnosis using nationally representative data (South Korean and Japanese populations), it did not meet the established eligibility criteria of our systematic review. The study analysed clusters of neuropsychiatric events according to nutritional status (Body Mass Index – BMI) rather than assessing specific symptoms individually, as required by our criteria (Supplementary Information, Tables S7 and S27). For instance, Tables S7 and S27 presented a stratified analysis of the long-term risk of neuropsychiatric events by BMI but did not report the risk for each of the 13 evaluated categories separately. To meet our eligibility criteria, the study would have needed to provide data for each category stratified by BMI, similar to the subgroup analyses in Tables S9 and S10, which assessed long-term hazard ratios (HR) for neuropsychiatric disorders according to COVID-19 severity and vaccination status. Due to variations in the symptom clusters analysed across studies, we excluded studies that examined clusters of symptoms according to nutritional status. Furthermore, the title/abstract of the study *“Short- and long-term neuropsychiatric outcomes in long COVID in South Korea and Japan”* did not mention *“excess weight,”* *“overweight,”* *“obesity,”* or *“body mass index.”* These terms represented **E**xposure in our predefined PECO framework and were included in our search strategy. Their absence from the title/abstract prevented the identification and retrieval of the study through our search. The exclusion criteria of our systematic review are detailed in the Methods section (lines 137–146), and the search strategy is outlined in the Methods section (lines 121–127) and Supporting Information (S1).

#4. Please describe GRADE system in method section in more detail.

Response: We appreciate the editor’s comments and have updated the Methods section of our study to provide a more detailed description of the GRADE system (lines 200-215).
